# Baicalein Acts against Candida albicans by Targeting Eno1 and Inhibiting Glycolysis

**DOI:** 10.1128/spectrum.02085-22

**Published:** 2022-07-28

**Authors:** Liping Li, Hui Lu, Xuan Zhang, Malcolm Whiteway, Hao Wu, Shanlun Tan, Jianye Zang, Shujuan Tian, Cheng Zhen, Xianlei Meng, Wanqian Li, Dazhi Zhang, Min Zhang, Yuanying Jiang

**Affiliations:** a Department of Pharmacy, Shanghai Tenth People’s Hospital, School of Medicine, Tongji University, Shanghai, China; b Department of Organic Chemistry, School of Pharmacy, Naval Medical University, Shanghai, China; c Hefei National Laboratory for Physical Sciences at Microscale, the First Affiliated Hospital of USTC, CAS Center for Excellence in Biomacromolecules, School of Life Sciences, University of Science and Technology of China, Hefei, Anhui, China; d Department of Biology, Concordia University, Montreal, Quebec, Canada; University of Iowa Hospitals and Clinics

**Keywords:** baicalein, Eno1, *Candida albicans*

## Abstract

Baicalein (BE) is a promising antifungal small-molecule compound with an extended antifungal spectrum, good synergy with fluconazole, and low toxicity, but its target protein and antifungal mechanism remain elusive. In this study, we found that BE can function against Candida albicans by disrupting glycolysis through targeting Eno1 and inhibiting its function. Eno1 acts as a key therapeutic target of the drug, as BE had no antifungal activity against the *eno1* null mutant in a Galleria mellonella model of C. albicans infection. To investigate the mechanism of action, we solved the crystal structure of C. albicans Eno1(CaEno1) and then compared the difference between this structure and that of Eno1 from humans. The predicted primary binding site of BE on CaEno1 is between amino acids D261 and W274, with D263, S269, and K273 playing critical roles in the interaction with BE. Both positions S269 and K273 have different residues in the human Eno1 (hEno1). This finding suggests that BE may bind selectively to CaEno1, which would limit the potential for side effects in humans. Our findings demonstrate that Eno1 is a target protein of BE and thus may serve as a novel target for the development of antifungal therapeutics acting through the inhibition of glycolysis.

**IMPORTANCE** Baicalein (BE) is a promising antifungal agent which has been well characterized, but its target protein is still undiscovered. The protein Eno1 plays a crucial role in the survival of Candida albicans. However, there are few antifungal agents which inhibit the functions of Eno1. Here, we found that BE can function against Candida albicans by disrupting glycolysis through targeting Eno1 and inhibiting its function. We further solved the crystal structure of C. albicans Eno1(CaEno1) and predicted that the primary binding site of BE on CaEno1 is between amino acids D261 and W274, with D263, S269, and K273 playing critical roles in the interaction with BE. Our findings will be helpful to get specific small-molecule inhibitors of CaEno1 and open the way for the development of new antifungal therapeutics targeted at inhibiting glycolysis.

## INTRODUCTION

Increased morbidity and mortality of invasive fungal infections are significant threats to immunocompromised patients (1). Currently, there are mainly three frontline clinical antifungal agent classes used to treat those infections, namely, polyenes, echinocandins, and azoles (2), and each of these classes has disadvantages that can limit their clinical application. Polyenes can cause severe side effects due to the structural similarity between target ergosterol and mammalian membrane sterol cholesterol (3). Although echinocandins have potent killing activity and an impressive safety profile, a limited antifungal spectrum, a requirement of intravenous administration, and high drug costs have become challenges for their clinical use (4, 5). Azoles have low toxicities and an extended antifungal spectrum but have only fungistatic effects in some species, which results in the emergence of azole-resistant isolates (3, 6). Therefore, the identification of new antifungal agents targeting novel targets is an urgent need for the management of invasive fungal infections.

Baicalein (BE) is a promising antifungal agent which has been well characterized. Previous studies have shown that BE has a broad antifungal spectrum and can significantly inhibit the growth of Aspergillus fumigatus, Cryptococcus neoformans, and *Candida* species (such as Candida albicans, Candida parapsilosis, Candida krusei, and Candida glabrata) (7–11). Furthermore, BE can suppress the formation of A. fumigatus and *Candida* species biofilms (12–15), which represent an essential mechanism for fungi to resist antifungal drugs. In addition, our previous study found that BE can enhance the efficacy of fluconazole (FLC) against FLC-resistant clinical isolates of C. albicans *in vitro* and transform FLC from fungistatic to fungicidal (16). Moreover, BE can induce apoptosis of C. albicans via the induction of reactive oxygen species (ROS) production (16–18). Although some target proteins of BE in bacteria have been reported, such as β-glucuronidase in Escherichia coli (19) and von Willebrand factor-binding protein in Staphylococcus aureus (20), the target protein of BE in fungi remains unclear.

The protein Eno1 (encoded by the *ENO1* gene [C1_08500C_A]) plays a crucial role in the survival, pathogenicity, and antifungal drug susceptibilities of C. albicans. First, Eno1 has enolase activity and plays an essential role in glycolysis (catalyzing the conversion of 2-phospho-d-glycerate [2-PG] to phosphoenolpyruvate) in C. albicans, as knocking out the *ENO1* gene renders C. albicans inviable on media with glucose as the sole carbon source (21, 22). Second, Eno1 has transglutaminase activity and an ability to bind host plasminogen (PLG) and thus plays a key role in the invasion and virulence of C. albicans (23–25). Furthermore, the homozygous deletion of the *ENO1* gene resulted in increased susceptibilities of C. albicans to frontline antifungal drugs, such as amphotericin B, FLC, miconazole, and voriconazole (21, 26). Thus, targeting Eno1 may be useful for treating C. albicans infection. However, there are few antifungal agents which inhibit the functions of Eno1.

In this study, we have determined that BE can bind to Eno1 and inhibit its enolase activity and that BE loses its antifungal effect when the *ENO1* gene is deleted, indicating that Eno1 is a therapeutic target of BE. Furthermore, we have determined the crystal structure of C. albicans Eno1(CaEno1); compared the difference between the crystal structure of Eno1 from C. albicans and that of humans; and identified the residues D263, S269, and K273 as important for the binding of BE to Eno1. This study reveals that Eno1 is the target protein of BE and might be a suitable target for the development of small-molecule drugs that could make a practical antifungal strategy for inhibiting glycolysis in the pathogen.

## RESULTS

### Carbon source requirement for BE activity.

**FIG 1:**
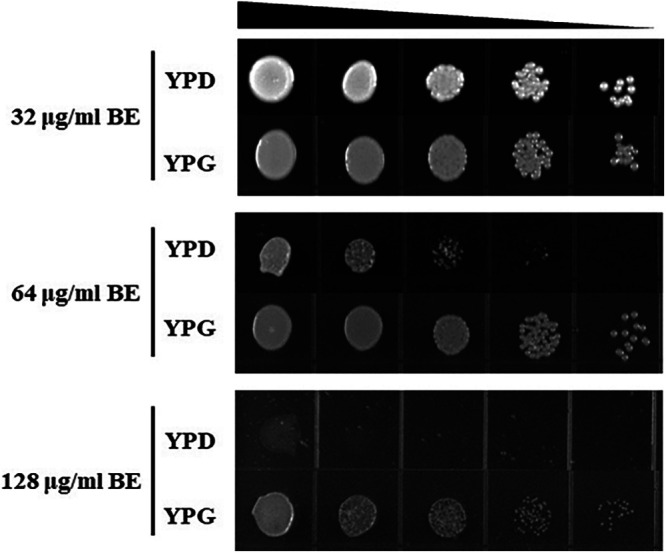
The antifungal activity of BE depends on the carbon source. The overnight-cultured C. albicans SC5314 cells were diluted to 1 × 10^7^ cells/mL and used as the initial concentration for four times of 10-fold dilution. Subsequently, 5 μL of a 10-fold dilution series was spotted onto YPD or YPG solid plates containing the indicated different concentrations of BE. After 48 h of incubation at 30°C, pictures of the growth of cells were taken.

To test whether BE antifungal activity in C. albicans as well as in mammalian cells was carbon source dependent (27), we investigated the activity of BE in spot assays on both solid yeast extract-peptone-dextrose (YPD) (containing 20 mg/mL glucose) and solid YPG (containing 2% [vol/vol] glycerol) media. The assay results showed that BE had antifungal activity against C. albicans at 64 μg/mL in solid YPD medium but not in solid YPG medium (Fig. 1). While BE at 128 μg/mL in the solid YPD medium had potent antifungal activity, C. albicans could still grow on a solid YPG medium with 128 μg/mL BE (Fig. 1). Thus, BE could inhibit C. albicans growth in medium containing glucose but not a nonfermentable carbon source (glycerol), suggesting a possible link between BE function and inhibition of glycolysis.

### Eno1 is a target protein of BE.

**FIG 2:**
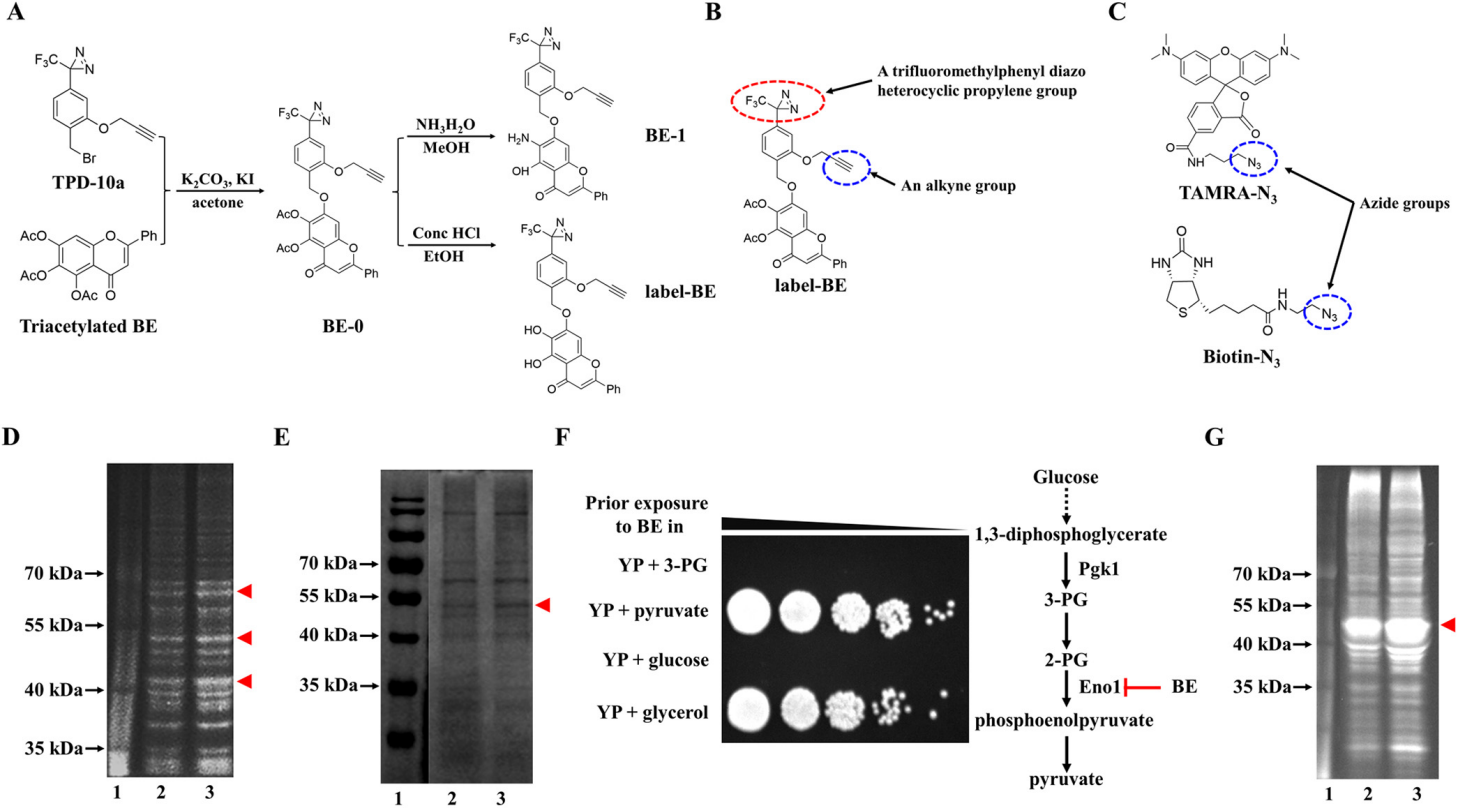
Eno1 is a target of BE. (A) Schematic diagram of synthesis label-BE. Label-BE is synthesized by intermediate TPD-10a with benzyl bromide structure and triacetylated BE. (B) The molecular structure of label-BE. Label-BE contains a trifluoromethylphenyl diazo heterocyclic propylene group (marked by a red dotted line) as a photoaffinity group to enhance the binding of label-BE to its target proteins and an alkyne group (marked by a blue dotted line) connected with an azide group (from the TAMRA-N3 or biotin-N3) by a copper-catalyzed azide-alkyne cycloaddition. (C) The molecular structures of TAMRA-N_3_ and biotin-N_3_. Both of them contain azide groups (marked by a blue dotted line) connected with an alkyne group of label-BE to highlight the target proteins of label-BE. (D) The competition experiment with BE and label-BE used the TAMRA-N3 as a reporter. C. albicans cell lysates were preincubated with 100 μM BE (lane 2) or not (lane 3). Subsequently, the two groups were incubated with 10 μM label-BE and irradiated with UV light at 365 nm and then treated with TAMRA-N3. After separation of SDS-PAGE, label-BE-targeted proteins were visualized by in-gel fluorescence scanning. The three fluorescent bands in lane 3 (indicated by red triangles) were cut out and pooled and then analyzed by LC-MS/MS. (E) The competition experiment with BE and label-BE used the biotin-N3 as a reporter. In the following enrichment with streptavidin-conjugated beads and separation by SDS-PAGE gel, targets of label-BE were visualized by Coomassie blue staining. The brighter band in lane 3 (indicated by red triangle) was then cut out and analyzed by LC-MS/MS. (F) BE has a potent antifungal activity in liquid YP medium with 3-PG and glucose. C. albicans cells were first treated with 128 μg/mL BE in liquid YP medium with either 3-PG (32 mg/mL), pyruvate (32 mg/mL), glucose (20 mg/mL), or glycerol (2% [vol/vol]) for 48 h and then were spotted (1:10 dilution) onto solid YPD medium and photographed after 48 h of incubation at 30°C. The red termination line represents inhibition; 3-PG, 3-phosphoglyceric acid. (G) The competition experiment with BE and label-BE used the TAMRA-N3 as a reporter by E. coli cell lysates containing recombinant C. albicans Eno1. Eno1 with a 6×His tag (about 50 kDa) in lane 3 (indicated by red triangle) and BE presence can remarkably reduce the fluorescent brightness of this band (lane 2).

We used to photoaffinity label to probe targets of BE. Biotin or fluorescent groups are used commonly to label molecules. Still, the probe molecules they label are usually large, and size may hinder the absorption of probe molecules by cells or affect the distribution of probe molecules within cells. It may also affect the affinity between probe and target. In this study, the probe compound binding the target protein and the reporter group highlighting the target protein were completed in two steps. Based on the parent structure of BE, we used the intermediate TPD-10a with a benzyl bromide structure and triacetylated BE as starting materials to obtain compound BE-0 and then derived BE-1 and label-BE, respectively, by ammonolysis and acidolysis (Fig. 2A; see Table S2 in the supplemental material). Label-BE had a synergistic activity with FLC against FLC-resistant C. albicans and thus can be used as an active probe for follow-up research (see Table S3 in the supplemental material). Label-BE contains a trifluoromethylphenyl diazo heterocyclic propylene group as a photoaffinity group (Fig. 2B). Through this photoaffinity group, label-BE was covalently combined with the target proteins by photolysis. Then the probe-protein complex was connected with the reporter groups (a rhodamine-azide conjugate [TAMRA-N_3_] and a biotin-azide conjugate [biotin-N_3_] were used in this study) by a copper-catalyzed azide-alkyne cycloaddition between an alkyne group in label-BE and an azide group in TAMRA-N_3_ or biotin-N_3_ (Fig. 2B and C; Table S2).

To avoid the effect of structural modification on the binding between compounds and proteins and to reduce the nonspecific binding between compounds and proteins, we carried out a target protein competition experiment with BE and label-BE to explore the target of BE. We divided C. albicans cell lysates into two groups; one group was preincubated with 100 μM BE, while the control group was not preincubated. Subsequently, the two groups were incubated with 10 μM label-BE and then treated with TAMRA-N_3_. After an SDS-PAGE gel analysis, we found three fluorescent bands significantly decreased in brightness (red triangles indicated bands in lane 2 in Fig. 2D) compared with the control group (lane 3 in Fig. 2D) due to BE’s competitive binding of those proteins which otherwise would be bound by label-BE. Therefore, potential target proteins of BE should be included in the three brighter bands of the control group. The three fluorescent bands in lane 3 in Fig. 2D were cut out and pooled and then analyzed by liquid chromatography-tandem mass spectrometry (LC-MS/MS). We found 30 proteins (score, >700; unique peptides, >10) in these 3 brighter bands (see Table S1A in the supplemental material); the target proteins of BE are expected to be included in these 30 proteins.

To verify the above result, we carried out another target protein competition experiment with BE and label-BE. In this case, the label-BE was tagged with a biotin-N_3_. As before, we divided the C. albicans cell lysates into two groups; one group was preincubated with 100 μM BE, and the other group was not preincubated. The two groups were then incubated with 10 μM label-BE and subsequently treated with biotin-N_3_. Following enrichment with streptavidin-conjugated beads, separation by SDS-PAGE gel, and staining by Coomassie blue, we found one band was reduced relative to the control group (a red triangle indicated the reduced band in lane 2 in Fig. 2E) due to BE competitively binding to proteins which should be bound by label-BE. Thus, potential target proteins of BE should be contained in the brighter band of the control group. The more brilliant band in lane 3 in Fig. 2E was then cut out and analyzed by LC-MS/MS, and 16 proteins (score, >700; unique peptides, >10) were identified explicitly (Table S1B). Eight proteins (AccI, Adh1, Eno1, Gnd1, Pdc11, Pgk1, Pyc2, and Tef1) were detected by both competitive experiments using label-BE tagged by TAMRA-N_3_ and biotin-N_3_. As mentioned above, since the antifungal activity of BE depends on inhibiting glycolysis, the two glycolysis-related proteins (Pgk1 and Eno1) were potential candidate targets of BE.

In order to further identify which glycolysis-related protein is the target of BE, we investigated the antifungal activity of BE in the media containing different intermediate products of glycolysis. 3-Phosphoglyceric acid (3-PG) is a downstream intermediate of Pgk1-catalyzed glycolysis but an upstream intermediate of Eno1-catalyzed glycolysis, while pyruvate is a downstream intermediate of Eno1-catalyzed glycolysis (Fig. 2F). C. albicans cells were first treated with 128 μg/mL BE in liquid YP medium with either 3-PG (32 mg/mL), pyruvate (32 mg/mL), glucose (20 mg/mL), or glycerol (2% [vol/vol]) for 48 h and then were spotted (1:10 dilution) on solid YPD medium and photographed after 48 h of incubation at 30°C. We found that there were no C. albicans cells growing after treatment with BE in liquid YP medium with 3-PG and glucose, indicating that BE had strong antifungal activity, but no antifungal activity in liquid YP medium with either pyruvate or glycerol (Fig. 2F). These results suggest that glucose and 3-PG need to be acted on by the target protein of BE to provide energy for C. albicans. In contrast, pyruvate and glycerol can give energy to C. albicans without the need for the target protein of BE. Therefore, we conclude that Eno1, but not Pgk1, is the target protein of BE.

To further test that Eno1 is the target protein of BE, we carried out a third competitive experiment between BE and label-BE using recombinant CaEno1. To obtain the recombinant Eno1, the full-length *ENO1* gene from C. albicans was cloned into the pET-21a (+) plasmid, generating pET-21a (+)-*ENO1*, and was then transformed into the E. coli strain BL21(DE3). Similar to previous competitive experiments, E. coli cell lysates containing recombinant CaEno1 were divided into two groups. One group was preincubated with 100 μM BE, and the other control group was not incubated. The two groups were then incubated with 10 μM label-BE and treated with TAMRA-N_3_. The widest and brightest band was CaEno1 with a 6×His tag (about 50 kDa) in line 3 in Fig. 2G, and BE presence can remarkably reduce the fluorescent brightness of this band (lane 2 in Fig. 2G). Our subsequent LC-MS/MS analysis of this bright band in line 3 in Fig. 2G showed that Eno1 was the dominant protein (Table S1C). This finding supports the model that BE and label-BE competitively bind to Eno1. Taken together, this information suggests Eno1 is a target protein of BE.

### BE has a specific binding activity to Eno1 and inhibits its enolase activity.

**FIG 3:**
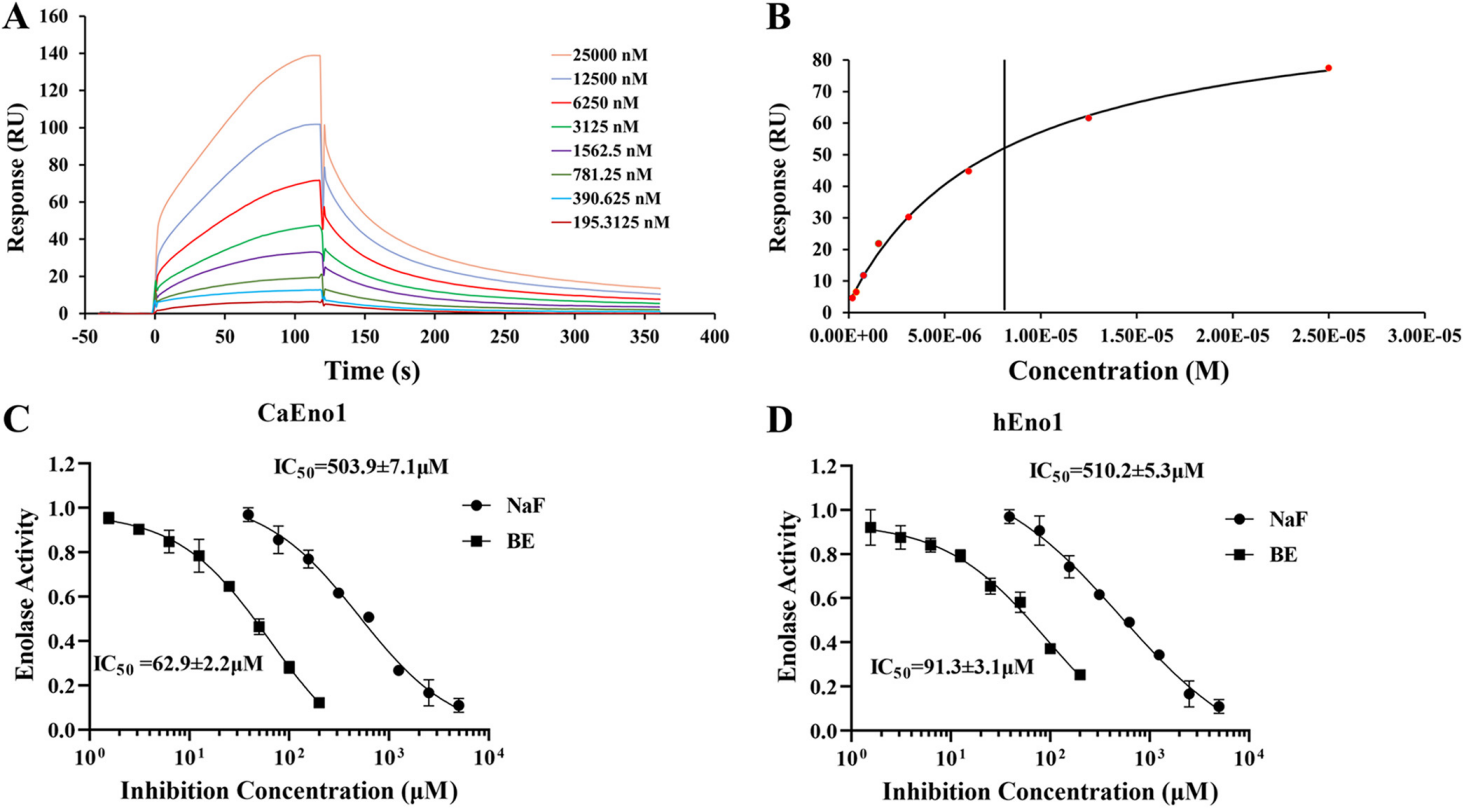
BE has a specific binding activity to Eno1 analyzed by SPR analyses and inhibits its enolase activity. (A) The kinetic analysis of SPR for binding curves (colored lines) was obtained by passing different concentrations of BE over Eno1 immobilized on a biosensor surface. SPR, surface plasmon resonance; RU, response unit. (B) The steady-state affinity analysis for the binding response at different concentrations of BE. A black line indicates the binding curve fitting data, and the vertical line indicates the binding affinity. (C) Inhibitory effect of BE on enolase of CaEno1. Data are shown as the means ± standard deviations. (D) The dose-dependent curve of BE inhibiting enolase activity Inhibitory effect of BE on enolase of hEno1. Data are shown as the means ± standard deviations.

We further carried out a surface plasmon resonance (SPR) assay to quantitatively evaluate the binding affinity of BE to Eno1. Briefly, the affinity constant (*K_D_*) between BE and Eno1 was determined by a Biacore T200 instrument. Eno1 was immobilized onto a Biacore CM5 sensor chip, and serial dilutions of BE were injected into the flow system for testing the binding affinity of BE to the Eno1. BE bound to the immobilized Eno1 in a slow on, slow off manner, despite the bulk response (Fig. 3A). We first conducted the regression of binding parameters with a kinetic model. The result showed a *K_D_* of 8.12 μM (Fig. 3A). In addition, it showed a moderate association rate constant (*k_a_* = 1,234 M^−1^s^−1^) and moderate dissociation rate constant (*K_d_* = 0.01002 s^−1^), which meant that BE could dissociate from Eno1 much slower than other typical small-molecule ligands, such as ENOblock, which also can bind to Eno1 (28). To test whether the fitting model could affect the binding affinity determination significantly, we also carried out the steady-state affinity analysis for the binding response (response unit [RU]) at different concentrations of BE. As shown in Fig. 3B, the binding curve fitting data were indicated by a black line, and the vertical line represented the binding affinity with a *K_D_* of 7.93 μM, shown in the lower table of detailed data (below, Fig. 3B). When the parameters determined from different models are compared, the binding constants are shown to be close to each other (8.12 μM in kinetic model versus 7.93 μM in the steady-state model), and the *R*_max_ values also did not change too much. We found that the theoretical stoichiometric ratio (SR) is around 1.03, which is close to 1 and indicates that BE binds specifically to CaEno1.

To test whether the interaction between BE and Eno1 affects the functioning of Eno1, we examined the inhibitory effect of BE on the enolase activity of CaEno1. The half-maximal inhibitory concentration (IC_50_) of BE inhibiting enolase activity is 62.9 ± 2.2 μM, which is significantly lower than that of the control compound NaF (503.9 ± 7.1 μM) (29) (Fig. 3C). These results suggested that BE can inhibit the enolase activity of Eno1, consistent with BE playing an antifungal role by inhibiting glycolysis (Fig. 1).

### Eno1 is a therapeutic target of BE.

**FIG 4:**
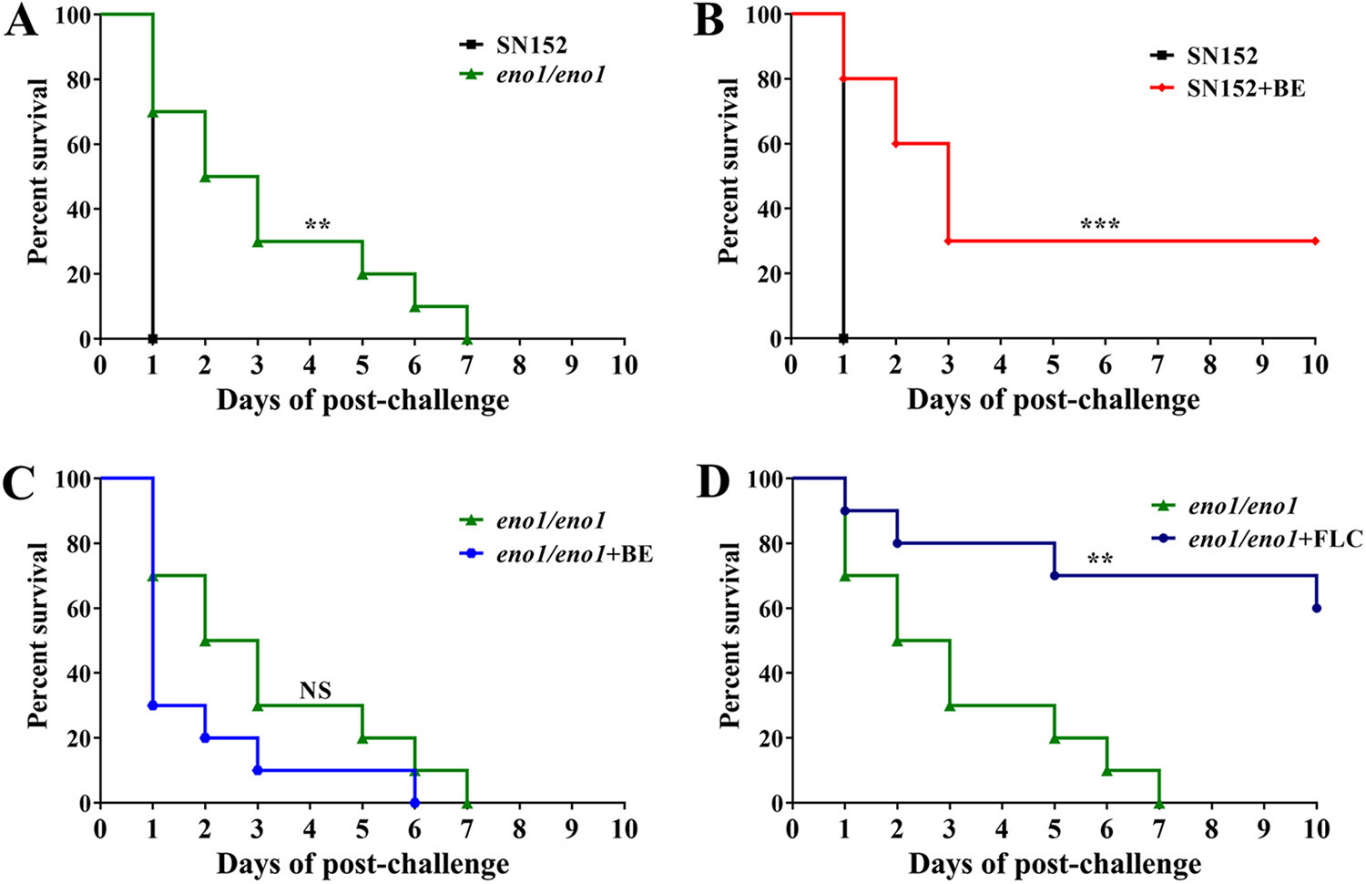
Eno1 acts as a therapeutic target of BE. G. mellonella larvae were injected with SN152 or the eno1/eno1 mutant strain (5 × 10^4^ cells/larvae) (A) followed by a second injection with either BE (B, C) or FLC (D). Survival curves of larvae infected with SN152 or the eno1/eno1 mutant (A); BE-treated, SN152 strain-infected group (B); BE-treated, eno1/eno1 mutant-infected group (C); and FLC-treated, eno1/eno1 mutant-infected group (D). Each curve represents a group of 10 larvae (*n* =10) monitored daily for survival for up to 10 days after infection. *P* values represent results of log-rank (Mantel-Cox) test comparing different treatment conditions with significance values as follows: ***, *P* < 0.001; **, *P* < 0.01; and NS, nonsignificant.

To assess whether Eno1 is a therapeutic target of BE, we constructed a homozygous deletion strain for the *ENO1* gene (*eno1/eno1*). We then investigated the difference in the antifungal efficacy of BE on the infections caused by the parent strain SN152 and the *eno1/eno1* strain in the Galleria mellonella infection model. We divided the G. mellonella into 5 groups, with 10 larvae in each group. All 10 larvae died after a 24-h infection with the SN152 strain, while only 3 larvae died after 24 h when infected with the *eno1/eno1* strain; the remaining larvae died within 7 days. Compared with the group infected with the parent strain, the survival time of the group infected with the *eno1/eno1* strain was longer (*P* < 0.01) (Fig. 4A). These results suggested that homozygous deletion of the *ENO1* gene led to the decrease of virulence of C. albicans, which is consistent with the previous study (21). The survival rate of larvae in the BE (10 mg/kg of body weight)-treated, SN152 strain-infected group was extended by 30%, compared with that in the group infected with the SN152 strain but without antifungal drug treatment (*P* < 0.001) (Fig. 4B). This result suggested that BE has antifungal activity *in vivo*. However, BE had no significant effect on the survival rate of G. mellonella infected by the *eno1/eno1* strain. All 10 larvae in the BE-treated group died within 6 days, and all 10 larvae without BE treatment were killed within 7 days (Fig. 4C). However, FLC (10 mg/kg) can extend the survival rate of larvae infected by the *eno1/eno1* strain by 60% (*P* < 0.01) (Fig. 4D) compared with the group infected with the *eno1/eno1* strain without antifungal drug treatment. These results indicated that the loss of the *ENO1* gene diminishes the susceptibility of C. albicans to BE but not to FLC, suggesting Eno1 is a therapeutic target of BE.

### Three-dimensional structure of CaEno1 and its difference from that of hEno1.

**FIG 5:**
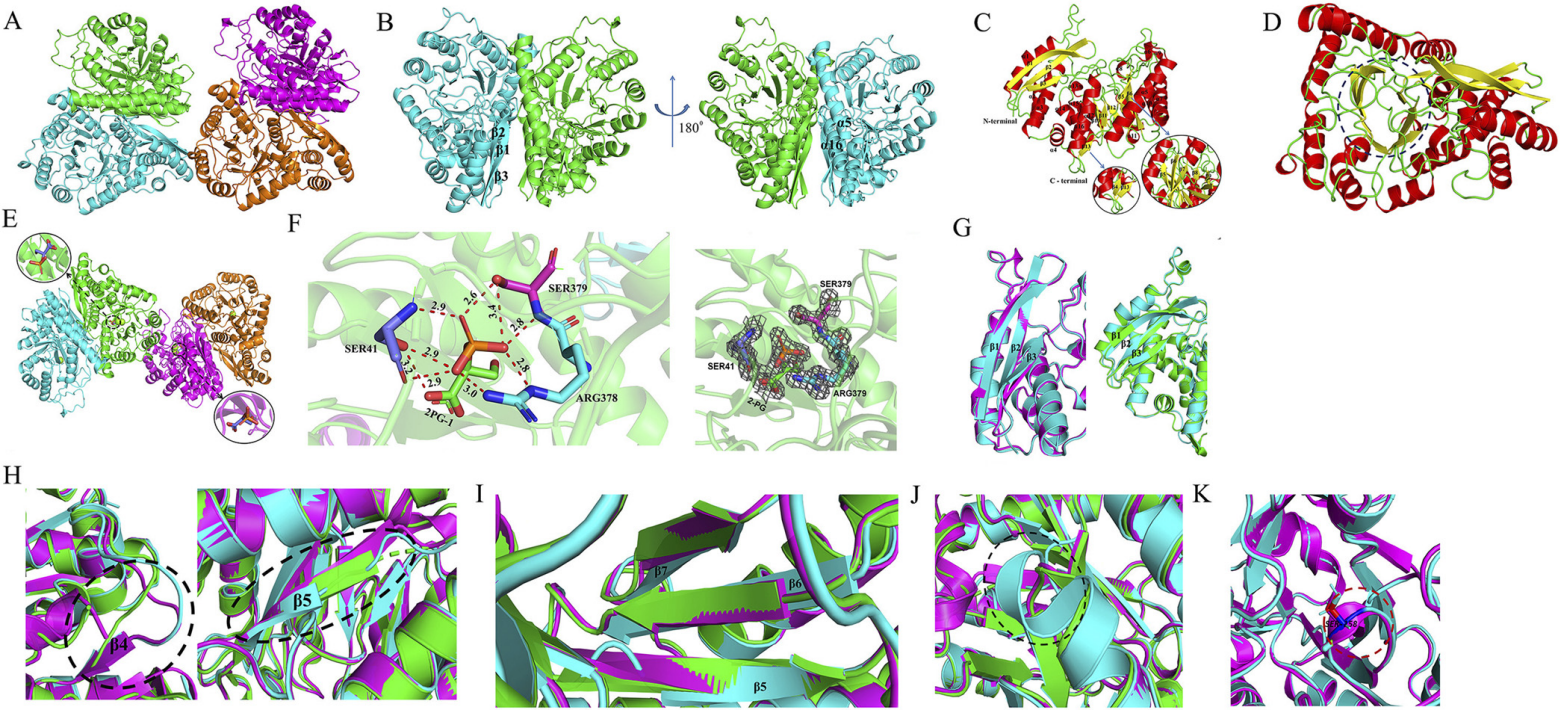
Three-dimensional structure of C. albicans Eno1 and its difference from that of S. cerevisiae Eno1 and human Eno1.(A) Crystal structure of C. albicans Eno1 tetramer. The interface formed by the two dimers is through four pairs of hydrogen bonds between the B chain (blue) and the D chain (orange). (B) Crystal structure of C. albicans Eno1 homodimer. In this dimer structure, the β1 to β3 strand plus the α5 helix and α16 helix of each Eno1 form an interaction interface. (C) The three-dimensional structure of a single Eno1 is composed of 13 β strands and 17 α helices. (D) The cavity formed by β strands is marked by a blue dotted line. (E) An asymmetric Eno1 tetramer binds to two 2-PG molecules in the A chain (green) and C chain (rose red). (F) Eno1 binds to 2-PG through nine hydrogen bonds, as follows: 2-PG forms four hydrogen bonds with S41 as both hydrogen bond donor and acceptor, with distances of 2.9 Å, 2.9 Å, 2.9 Å, and 3.2 Å; as a hydrogen bond donor, 2-PG forms two hydrogen bonds with R378 at distances of 2.8 Å and 3.0 Å; and 2-PG forms three hydrogen bonds with S379 as hydrogen bond donor and acceptor, and their distances are 2.4 Å, 2.8 Å, and 3.4 Å. 2-PG, 2-phospho-d-glycerate (left); the 2Fo-Fc electron-density map of 2-PG (right). (G) The differences among β1 to β3 strands of C. albicans Eno1 (blue) and that of S. cerevisiae Eno1 (purple) and human Eno1 (green). (H) In the region of S139 to F146, C. albicans Eno1 and S. cerevisiae Eno1 contain a β2 strand, but there is no β strand in human Eno1 (left); the difference of F152 to Q153 leads to the length of the β5 strand of C. albicans Eno1 being shorter than that of S. cerevisiae Eno1 and human Eno1 (right). (I) The difference of amino acids F152 to Q153, F168, and G244 leads to significant differences in the structure of antiparallel β5-β6-β7. (J) The length of the α12 helix of C. albicans Eno1 (blue) is longer than that of S. cerevisiae Eno1 (purple) and human Eno1 (green) due to the difference of D317 to K318. (K) Around amino acid S158, there is an α helix in S. cerevisiae Eno1 (purple) and human Eno1 (green) but no α helix in C. albicans Eno1 (blue).

We established the X-ray structure of full-length CaEno1 in complex with 2-PG (PDB 7V67) at a resolution of 2.3 Å. The spatial group of the crystal structure of CaEno1 is C2, and the *R*_factor_/*R*_free_ is 17.69/21.83. A total of 98.20% of the amino acid residues are in the optimal region and 1.68% are in the generally acceptable region of the Laplace diagram (Table 1). The diffraction data show that in an asymmetric unit, four Eno1 proteins form two dimers. The interface formed by the two dimers is through four pairs of hydrogen bonds, as follows: K80 in the B chain and E285 and R314 in the D chain form two hydrogen bonds, D72 in the B chain and Q286 in the D chain form one hydrogen bond, and S425 in the B chain forms one hydrogen bond with K202 in the D chain (Fig. 5A). Two CaEno1 molecules form a homodimer face-to-face in an antiparallel manner. In this dimer structure, the β1 to β3 strand plus the α5 helix and α16 helix of each Eno1 form an interaction interface (Fig. 5B). The three-dimensional structure of a single Eno1 is composed of 13 β strands and 17 α helices, in which the N-terminal domain (1 to 138) contains 4 α helices (α1 to α4 helix) and 1 antiparallel β sheet (β1 to β3 strand), while the C-terminal domain (138 to 439) consists of 13 α helices (α5 to α17 helix), 2 antiparallel β sheets (the β5 to β9 strand forms 1 and the β10 to β12 strand forms another), and 1 parallel β sheet (β4 strand and β13 strand). The β1 to β3 strand is exposed to the protein surface, and the rest of the β strands are embedded in the interior of Eno1 (Fig. 5C). Interestingly the β4 to β13 strand forms a large β-barrel, while the α helices of the C-terminal domain are wrapped in the β-barrel to form a mixed α/β-barrel (Fig. 5D). This structure is similar to the three-dimensional structure of Eno1 in other species, suggesting that the active site of CaEno1 may also be in the C-terminal domain. An asymmetric Eno1 tetramer binds to two 2-PG molecules in the A chain and C chain through nine hydrogen bonds (PDB 7VRD) (Table 1), as follows: 2-PG forms four hydrogen bonds with S41 as both the hydrogen bond donor and acceptor, with distances of 2.9 Å, 2.9 Å, 2.9 Å, and 3.2 Å; as a hydrogen bond donor, 2-PG forms two hydrogen bonds with R378 at distances of 2.8 Å and 3.0 Å; and 2-PG forms three hydrogen bonds with S379 as the hydrogen bond donor and acceptor, and their distances are 2.4 Å, 2.8 Å, and 3.4 Å (Fig. 5E and F, left). The 2Fo-Fc electron density map of 2-PG and the key amino acid residues were contoured at 1.0 σ (gray) (Fig. 5F, right).

**TABLE 1:** Data collection and refinement statistics

Parameter	Data by PDB	
	7V67	7VRD
Data collection		
Space group	*C2*	*C2*
Cell dimensions		
*a*, *b*, *c* (Å)	265.20, 62.18, 111.82	264.26, 61.85, 111.75
α, β, γ (°)	90, 109.91, 90	90, 109.80, 90
Resolution (Å)^*a*^	33.10- 2.00 (2.07–2.00)	38.06-1.70 (1.76–1.70)
*R*_sym_ (%)^*b*^	3.5 (23.4)	6.9 (50.0)
Overall I/σ (I)	12.79 (3.48)	23.3 (3.0)
Completeness (%)	99.7 (99.86)	99.6 (98.9)
Redundancy	6.6 (5.0)	6.7 (6.9)
Reflections (Unique)	116,013 (11,527)	1,237,061 (185,469)
CC1/2	0.998 (0.853)	0.999 (0.921)
Refinement		
*R*_factor_^*c*^/*R*_free_^*d*^	17.69/21.83	17.20/19.66
RMS deviations		
Bond lengths (Å)	0.007	0.0065
Bond angles (°)	0.83	1.17
No. of atoms		
Protein	13,156	13,278
Ligand		22
Water	398	1,205
B-factor (Å^2^)		
Protein	28.86	18.69
Ligand		13.47
Water	27.52	25.31
Ramachandran plot^*e*^		
Most favored regions (%) (%)	98.20	98.68
Additionally allowed (%) (%)regions (%)	1.68	1.09
Outliers (%)	0.12	0.23

a The values in parentheses refer to statistics in the highest shell.

b Rsym = |Ii−|/|Ii| where Ii is the intensity of the ith measurement and is the mean intensity for that reflection.

c Rfactor = ∑∑hkl‖Fo‖Fc‖/∑∑hkl|Fo|, where |Fo| and |Fc| are the observed and calculated structure factor amplitudes, respectively.

d *R*_free_ was calculated with 5.0% of the reflections in the test set.

e Statistics for the Ramachandran plot from an analysis using MolProbity.

We compared the sequence and structure of CaEno1 with S. cerevisiae Eno1 and hEno1. The consistency of amino acid sequences of CaEno1 versus S. cerevisiae Eno1 and CaEno1 versus hEno1 were 76.66% and 61.98%, respectively (see Fig. S1 in the supplemental material). Although the overall three-dimensional structure of CaEno1 was similar to that of S. cerevisiae Eno1 and hEno1, because the root mean square deviation (RMSD) of CaEno1 and S. cerevisiae Eno1 (PDB 1One) was 0.395 Å (see Fig. S2A in the supplemental material) and that of CaEno1 and hEno1 (PDB 3B97) was 0.418 Å (Fig. S2B), there were some noteworthy differences, which are as follows. (i) The length of the β1 strand and β2 strand in CaEno1 is longer than that of S. cerevisiae Eno1 and hEno1 (Fig. 5G), which is due to the difference between T5, 8H, Y11, F25, and T26. (ii) The length of the β3 strand of CaEno1 is shorter than that of S. cerevisiae Eno1 and hEno1, which may be due to the difference of D28 and S34 in the amino acid sequence (Fig. 5G). (iii) In the region of S139 to F146, in contrast to CaEno1 and S. cerevisiae Eno1, which contain a β2 strand, there is no β strand in hEno1 (Fig. 5H, left). (iv) The difference of F152 to Q153 leads to the length of β5 strand of CaEno1 being shorter than that of S. cerevisiae Eno1 and hEno1 (Fig. 5H, right). (v) The difference of amino acids F152 to Q153, F168, and G244 leads to significant differences in the structure of antiparallel β5-β6-β7 (Fig. 5I). (vi) The length of the α12 helix is longer than that of S. cerevisiae Eno1 and hEno1 due to the difference of D317 to K318K (Fig. 5J). (vii) Around amino acid 158S, there is an α helix in S. cerevisiae Eno1 and hEno1 but no α helix in CaEno1 (Fig. 5K).

### A potential binding site of BE in Eno1 is D261 to W274.

**FIG 6:**
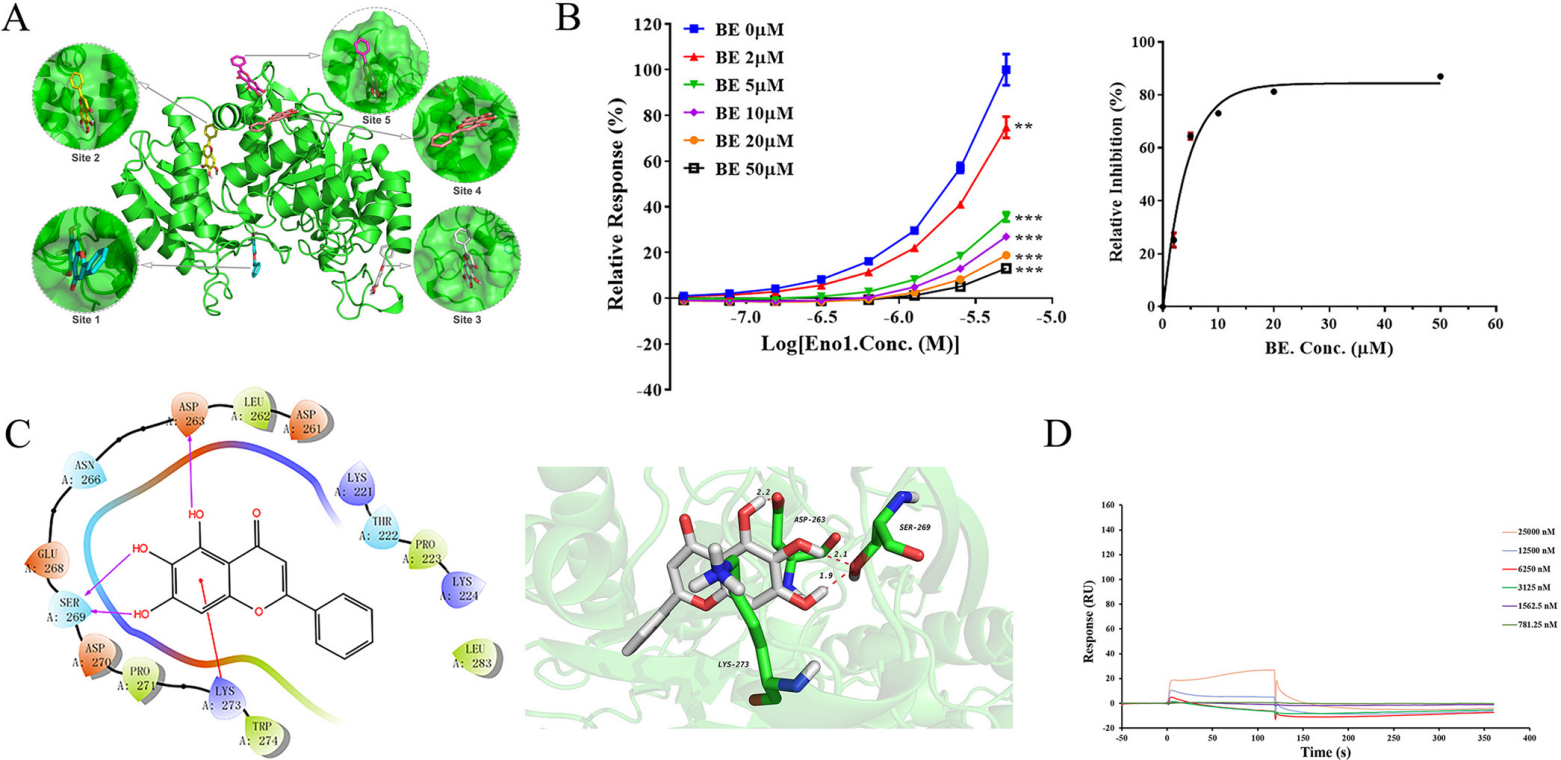
The predicted primary binding site of BE on C. albicans Eno1 is D261 to W274. (A) Five potential binding sites of BE on Eno1 are as follows: site 1 (residues 37 to 51), site 2 (residues 133 to 147), site 3 (residues 384 to 389), site 4 (residues 138 to 142, 394 to 395, and 438 to 439), and site 5 (residues 261 to 274). BE has the strongest binding interaction with site 5 (docking score, −5.703). (B) BE can significantly inhibit the interaction of Eno1 and PLG by SPR analysis. PLG (50 μg/mL) was coated on the CM5 sensor chip and incubated with different concentrations of Eno1 in the presence of BE in indicated concentrations. Relative inhibitions (%) obtained a response to different concentrations of BE were used to yield the fitting curve and calculate the IC_50_ value for the inhibitory effect of BE on the interaction of Eno1 and PLG. ***, *P* < 0.001; **, *P* < 0.01. (C) BE is modeled to bind to Eno1 with three hydrogen bonds (two hydrogen bonds with S269 at distances of 1.9 Å and 2.1 Å; a hydrogen bond with D263 as a hydrogen bond donor at a distance of 2.2 Å), cation-π interaction (with K273), and hydrophobic interaction. (D) The kinetic analysis of SPR for binding curves (colored lines) obtained by passing different concentrations of BE over mutant Eno1 (D263A, S269A, and K273A) immobilized on a biosensor surface. SPR, surface plasmon resonance; RU, response unit.

To identify the structural determinants for BE binding of Eno1, we tried to cocrystallize Eno1 with BE. However, due to the poor diffraction of BE-bound Eno1 crystals caused by the poor solubility of BE and the low affinity of BE for Eno1, the structural determination of the ligand complex was not possible. As an alternative, we investigated the Eno1-BE complex by molecular docking using the Glide method. We identified five potential binding sites of BE on Eno1, as follows: site 1 (residues 37 to 51), site 2 (residues 133 to 147), site 3 (residues 384 to 389), site 4 (residues 138 to 142, 394 to 395, and 438 to 439) and site 5 (residues 261 to 274). The docking scores of BE with site 1, site 2, site 3, site 4, and site 5 were −4.047, −5.205, −4.109, −4.391, and −5.703, respectively (Fig. 6A). The docking score of the Schrödinger software is negative—the higher the absolute value, the stronger the binding force between the compound and protein. From the docking score, BE has the strongest binding interaction with site 5, which is located at flexible ring L3 (residues 251 to 277), which is one of three-ring regions (L1 [residues 37 to 44], L2 [residues 169 to 171], and L3) closely related to enolase activity (similar to S. cerevisiae structure) (30). In addition, because BE can significantly inhibit the interaction of Eno1 and PLG, the IC_50_ of BE inhibiting the interaction between Eno1 and PLG was 2.95 μM (Fig. 6B). The binding site of PLG in CaEno1 is F254 to D263 through the alignment of Eno1 amino acid sequences of several microorganisms (see Fig. S3 in the supplemental material) (31–37), and the most likely binding site of BE in Eno1 is site 5 (D261 to W274).

In site 5, BE is modeled to bind to Eno1 with three hydrogen bonds, a cation-π interaction, and a hydrophobic interaction; two hydroxyl groups (C-6 and C-7) as hydrogen bond donors form two hydrogen bonds with S269 at distances of 1.9 Å and 2.1 Å, respectively; The other hydroxyl group (C-5) forms a hydrogen bond with D263 as a hydrogen bond donor at a distance of 2.2 Å. The benzene ring with hydroxyl group can form a cation-π interaction with K273 (Fig. 6C). To confirm that D263, S269, and K273 play crucial roles in interacting with BE, we have mutated these three amino acids (D263A, S269A, and K273A) in CaEno1. We found no binding between BE and the mutant Eno1 detected by SPR analysis (Fig. 6D), indicating that D263, S269, and K273 contribute to the interaction between BE and CaEno1. Besides, aligning amino acid sequences of CaEno1 versus hEno1, we found that S269 and K273 from site 5 are different between CaEno1 and hEno1 (Fig. S1). We found that the IC_50_ of BE inhibiting the enolase activity of hEno1 was 91.3 ± 3.1 μM (Fig. 3D), which was moderately weaker than that of BE inhibiting enolase activity of CaEno1 (*P* < 0.5). This result suggests that BE may selectively bind to CaEno1, which would limit the potential for side effects in humans.

## DISCUSSION

In this study, we propose that BE has anti-C. albicans activity by targeting Eno1 and by consequently inhibiting glycolysis (Fig. 1 and Fig. 2D to G). BE has targeted binding to Eno1 as tested by SPR analyses (Fig. 3A and B), and BE can significantly inhibit the enolase activity of CaEno1 (Fig. 3C). These results support the potential of antifungal strategies directed at inhibiting glucose metabolism. Glucose can affect the ability of C. albicans cells to transit from yeast to hyphae, to form a biofilm, and to generate resistance to oxidative stress and antifungal drugs (38–43). Therefore, inhibiting the metabolic utilization of glucose, such as inhibiting glycolysis, can be a promising antifungal strategy, just as glucose limitation has been a profitable strategy for anticancer therapy (44). It has been reported that SF2312 and its analogues have antitumor activity as enolase inhibitors (45, 46), but few antifungal substances specifically inhibit this enzyme. Enolase is a dimeric enzyme and is critical for glycolysis (Fig. 5B), where it catalyzes the conversion of 2-PG to phosphoenolpyruvate. Our findings demonstrate that BE can target Eno1 and consequently inhibit glycolysis, which makes the strategy of antifungals through inhibiting glucose metabolism practicable. Beyond that idea, according to our findings and previous findings that inferred enolase activity can induce ROS production (47), we can potentially clarify the mechanism of BE-induced ROS production in C. albicans. Our previous studies have found that BE can induce apoptosis of C. albicans cells through hyperproduction of ROS (16–18), but its mechanism is not clear. We propose that when BE inhibits enolase activity, it will lead to increased glucose uptake, resulting in excess intracellular glucose being forced into the polyol pathway, which metabolizes glucose into sorbitol and NADH (47). Activation of aldose reductase, the key enzyme of the polyol pathway, increases the consumption of NADPH (48), in turn, to activate the pentose phosphate pathway, which promotes NADPH oxidase activation by restoring NADPH (47). Finally, the activated NADPH oxidase contributes to the higher ROS levels of BE-treated C. albicans cells (49).

We found that Eno1 is a therapeutic target of the antifungal effect of BE (Fig. 4A to C). In the present study, Eno1 is identified as a target of BE (Fig. 2F and G), and its enolase activity can be inhibited by BE (Fig. 3C). Consequently, BE has powerful antifungal activity in media containing glucose (Fig. 1). Furthermore, we found that BE has no antifungal effect in the *eno1/eno1* strain-infected Galleria mellonella model (Fig. 4C). Therefore, BE may bind to various proteins, but BE plays an antifungal role by targeting Eno1 (Fig. 3A and B) and inhibiting its functions (Fig. 3C and Fig. 6B).

We obtained the crystal structure of CaEno1 (Fig. 5A to C); compared the difference between the crystal structure of Eno1 from C. albicans and humans (Fig. 5G to K); predicted the most likely binding site of BE in Eno1 is D261 to W274 (Fig. 6A and C); and found D263, S269, and K273 play a crucial role in the interaction between BE and Eno1 (Fig. 6D). A conserved Eno1 amino acid sequence is the major challenge in screening small-molecular antifungal compounds that selectively inhibit fungal Eno1. And although BE is a promising antifungal compound (12, 16–18), the structure of BE also needs to be further optimized to obtain better antifungal activity *in vitro* and *in vivo*. However, due to the previously unclear target protein of BE, no significant progress has been made. Our findings will be helpful for identifying specific small-molecule inhibitors of CaEno1 by using virtual screening and computer-aided design in the future. These findings will be beneficial to optimize the structure-activity of BE to obtain its analogues with better antifungal activity.

In conclusion, in the present study, we established that Eno1 is a therapeutic target of the antifungal activity of BE and obtained the crystal structure of CaEno1. These findings will be helpful to encourage specific small-molecule inhibitors of CaEno1 and open the way for the development of new antifungal therapeutics targeted at inhibiting glycolysis.

## MATERIALS AND METHODS

### Strains, primers, agents, and culture conditions.

All strains and primers used in this study are listed in Table S2A and S2B, respectively. *Candida* strains were cultured routinely at 30°C in either YPD media (1% yeast extract, 2% peptone, and 2% dextrose) or YPG media (1% yeast extract, 2% peptone, and 2% glycerol). Medium plates were supplemented with 2% agar. Drug stock solutions were prepared using dimethyl sulfoxide (DMSO) as a solvent for BE (6.4 mg/mL) (Aladdin) for the spotting assay and SPR analysis and for TAMRA-N3 (5.123 mg/mL) (synthesized in this study) and biotin-N3 (3.123 mg/mL) (synthesized in this study) for competition experiments; using phosphate-buffered saline (PBS) as a solvent for FLC (1.0 mg/mL) and BE (1.0 mg/mL) for Galleria mellonella virulence assays; using 80% DMSO-20% Tween 80 as a solvent for BE (6.4 mg/mL) for competition experiments and microscale thermophoresis (MST) analysis and label-BE (2.728 mg/mL) (synthesized in this study) for competition experiments; and using sterilized double distilled water as a solvent for PLG (1.0 mg/mL) (Sigma-Aldrich), 3-PG (128 mg/mL) (Shyuanye, China), and sodium pyruvate (128 mg/mL) (Sangon Biotech, China). Once in solution, drugs were stored at −40°C.

### Spotting assay.

The spotting assay was performed as described previously (50). Briefly, after grown overnight, mid-log-phase cultures of strains required for experimental conditions were adjusted to 1 × 10^7^ cells/mL using a hemacytometer, then were 1:10 serially diluted in sterile PBS, and were spotted onto indicated plates with an indicated concentration of BE. After 48 h of incubation at 30°C, pictures of the growth of cells were taken.

### Expression and purification of recombinant CaEno1 and hEno1.

To obtain recombinant CaEno1, the C. albicans *ENO1* coding sequence was amplified with primers ENO1up/ENO1down, digested with BamHI/XhoI, and cloned into the BamHI/XhoI-digested pET-21a(+) plasmid, resulting in plasmid pET-21a(+)-*ENO1*, which was sequenced and transformed into E. coli strain BL21(DE3) (Tiangen, China). A single colony of BL21(DE3) transformed with the plasmid pET-21a (+)-*ENO1* was incubated in 50 mL of LB media containing 100 μg/mL ampicillin at 37°C for 14 h shaking at 200 rpm. The E. coli cultures were transferred into freshly prepared 1 L LB medium with 100 μg/mL ampicillin and incubated to an optical density at 600 nm (OD_600_) of 0.6 to 0.8. Then the culture was induced with 0.4 mM isopropyl-β-d-thiogalactopyranoside (IPTG), and the incubation was further carried out for 22 h at 16°C. Cells were harvested by centrifugation at 6,000 × *g* for 8 min at 4°C and resuspended in lysis buffer (50 mM Tris-HCl [pH 7.5], 500 mM NaCl, 5% [vol/vol] glycerol, 2 mM β-mercaptoethanol [β-ME], and 1 mM phenylmethylsulfonyl fluoride [PMSF]). The cells were then homogenized by sonication, and the lysate was centrifuged at 23,800 × *g* for 30 min at 4°C. The resulting supernatant containing the target protein fused with the 6×His tag was further subjected to Ni-nitrilotriacetic acid (NTA) (Qiagen) affinity chromatography columns pre-equilibrated with lysis buffer and incubated for 30 min at 4°C. The columns were washed with 50 column volumes of washing buffer (50 mM Tris-HCl [pH 7.5], 500 mM NaCl, 5% [vol/vol] glycerol, 2 mM β-ME, and 60 mM imidazole) to remove contaminants, and the bound proteins were eluted with 3 column volumes of elution buffer (50 mM Tris-HCl [pH 7.5], 500 mM NaCl, 5% [vol/vol] glycerol, 2 mM β-ME, and 250 mM imidazole). The eluted proteins were further purified using a Superdex 200 increase size exclusion column (GE Health Care) pre-equilibrated with size exclusion chromatography (SEC) buffer (20 mM Tris [pH 7.5], 200 mM NaCl, and 1 mM dithiothreitol [DTT]). SDS-PAGE assessed the peaks corresponding to target proteins.

To obtain recombinant hEno1, we synthesized and optimized the full-length sequence of the human alpha-enolase (UniProt accession no. P06733) coding sequence (Tsingke Biotechnology Company). The recombinant hEno1 was expressed and purified using the same procedure as that of CaEno1.

### Synthesis of label-BE, TAMRA-N_3_, and biotin-N_3_.

The detailed procedures of the synthesis of label-BE, TAMRA-N_3_, and biotin-N_3_ can be found in Text S1 in the supplemental material. The compounds were identified by nuclear magnetic resonance (NMR) (Bruker DRX-600) (see Table S4 in the supplemental material).

### Extraction of total protein from C. albicans.

A single clone of C. albicans was inoculated in 1 mL YPD liquid medium at 200 rpm at 30°C for 16 h. Next, 1 mL of C. albicans overnight cultures was inoculated into 100 mL of fresh YPD liquid medium and placed onto a shaking table at 30°C at 200 rpm. They were cultured for 16 h and centrifuged at 4°C at 3,000 rpm for 5 min, the supernatant was discarded, and C. albicans cells were collected. The cells were washed three times with sterilized PBS (pH 7.4) and centrifuged at 3,000 rpm at 4°C for 5 min. A total of 10 mL of protein extract buffer (50 mm Tris, 1.5 mm EDTA, 1% Triton X-100, and 0.4% SDS [pH 7.5]) was added, and glass beads at 2 times the total volume were added. The mixture was transferred to the bead beater instrument (Hamilton Beach Brands, Inc.) and maintained at low temperature (ice bath conditions) for 30 s at an interval of 30 s, and this procedure was repeated 10 times. The C. albicans cell lysates were removed and centrifuged at 13,000 rpm at 4°C for 5 min, and the supernatant was isolated, including the total protein solution, which was stored at −80°C.

### Target protein competition experiments.

Target protein competition experiments between BE and label-BE were performed as described previously (51, 52). Briefly, C. albicans cell lysates were split into two groups; each group contained 500 μL of protein solution (1.3 mg/mL). One group was preincubated with 100 μM BE, and the other group without BE was a control group. Then the two groups were incubated with 10 μM label-BE and allowed to bind its protein targets. After UV irradiation at 365 nm, TAMRA-N3 or biotin-N3 was then added and coupled to the small alkyne handle of the label-BE using the copper-mediated click chemistry reaction. Subsequently, for labeling label-BE with TAMRA-N_3_, protein solutions were separated by an SDS-PAGE gel. The bands with changes in fluorescence brightness were then cut off and analyzed by LC-MS/MS; for labeling label-BE with biotin-N_3_, protein solutions were enriched with streptavidin-conjugated beads. Protein targets were then eluted by 6 M urea and separated by an SDS-PAGE gel. The bands with brightness change on the SDS-PAGE gel were cut off and analyzed by LC-MS/MS. A competitive experiment between BE and label-BE using recombinant CaEno1 was carried out according to the method of the above competitive experiment, in which label-BE was labeled with TAMRA-N_3._

### Surface plasmon resonance (SPR) analysis.

SPR analysis was performed with a Biacore T200 instrument (GE Healthcare) with CM5 sensor chips (GE Healthcare) as described previously (28). Activation, deactivation, preparation of the coupled flow cell, and the ligand-binding assay were performed as described previously (53). Briefly, Eno1 was immobilized in parallel-flow channels on a Biacore CM5 sensor chip using an amine coupling kit (GE Healthcare). For testing the binding of Eno1 by BE, serial dilutions of BE (25,000 nM, 12,500 nM, 6,250 nM, 3,125 nM, 1,562.5 nM, 781.25 nM, 390.625 nM, and 195.3125 nM) were injected into the flow system. Experiments were conducted with PBS as the running buffer, and the analyte was injected at a flow rate of 30 μL/min. The association time was 90 s and the dissociation time was 60 s. The binding affinity constants were obtained using a 1:1 Langmuir binding model via BIA evaluation software. We also used SPR analysis to test the ability of BE to inhibit the interaction of Eno1 and PLG using SPR analysis. Briefly, PLG (50 μg/mL) was first coated onto a CM5 chip and then incubated with different concentrations of Eno1 (5 μM, 2.5 μM, 1.25 μM, 0.625 μM, 0.3125 μM, 0.15625 μM, 0.078 μM, and 0.039 μM) in the presence or absence of BE (50 μM, 20 μM, 10 μM, 5 μM, 2 μM, and 0 μM), followed by the above steps, and finally analyzed by BIA evaluation software.

### Enolase activity analysis.

Enolase activity was determined by direct spectrophotometric assay via measuring the increase of absorbance at 240 nm of phosphoenolpyruvate (PEP) as described previously with some modifications (54). Briefly, the reaction buffer (pH 7.0) containing 10 mM imidazole, 200 mM KCl, and 0.5 mM MgAc in a final reaction volume of 100 μL was mixed with Eno1 (a final concentration of 30 nM), followed by the mixture of 2-phosphoglycerate (2-PG) (Yuanye Biotech Shanghai) with a final concentration of 1 mM. The enolase activity of Eno1 was evaluated by measuring the increase of absorbance (OD_240_) at room temperature for 10 min. For the inhibition study of enolase by BE, different concentrations of BE were used to mix well with Eno1 and were incubated at room temperature for 5 min, and subsequent operations were described above. In the final reaction volume of 100 μL of this assay, the initial concentration of BE was 200 μM and serially diluted 2-fold for eight different concentrations. The well-known enolase inhibitor sodium fluoride (NaF; Sangon Biotech) was initiated from 5 mM and used as a control (29). Inhibition curves were fitted by nonlinear regression (four parameters) using the GraphPad Prism software.

### Disruption of the *ENO1* gene.

The two alleles of the *ENO1* gene (C1_08500C_A) were deleted from the strain SN152 using a fusion PCR method as described previously (55). The first round of reactions involves amplification of the flanking sequences of the *ENO1* gene (with a template of genomic DNA and primers ENO1P1 and ENO1P3 or ENO1P4 and ENO1P6, in separate reactions) and the selectable marker (*HIS1* or *ARG4*) (with a template of plasmid pSN52 or pSN69 and primers universal primer 2 and universal primer 5). The 5′ tails of universal primer 2 and ENO1P3 are complementary, as are the 5′ tails of ENO1P4 and universal primer 5. In the fusion round of PCR, all three products of the first round are combined, and a fusion product is amplified with primers ENO1P1 and ENO1P6. The transformation was carried out according to protocols of the Yeastmaker yeast transformation system 2 kit (Clontech Laboratories, Inc.) and transfected colonies selected on synthetic media (2% dextrose, 6.7% yeast nitrogen base without amino acids, and 2% agar) containing the necessary auxotrophic supplements for the heterozygous *ENO1*/*eno1* mutant strain and on synthetic media (2% glycerol, 6.7% yeast nitrogen base without amino acids, and 2% agar) containing the necessary auxotrophic supplements for the homozygous *eno1*/*eno1* null mutant strain. The primers used to diagnose the *ENO1* gene knockouts were, for the 5′ junctions, a primer ENO1UCheck plus a primer HIS1left or ARG4left, as appropriate; and for the 3′ junctions, a primer ENO1Dcheck plus a primer HIS1right or ARG4right.

### Galleria mellonella virulence assays.

Virulence testing of C. albicans was done in Galleria mellonella as described previously (56). Larvae were selected randomly for experiments in groups of 5, and those showing signs of melanization were excluded. Larvae with an average weight of 500 mg were cultured at 37°C overnight before the experiment in each group. After growth in YPD media overnight at 30°C and 200 rpm, C. albicans SN152 cells or *eno1*/*eno1* mutant cells were washed with PBS 3 times and diluted to the desired concentration (1 × 10^7^ cells/mL) determined by counting with a hemacytometer. Larvae were infected with 5 μL of a PBS suspension of 1 × 10^7^ cells/mL using a Hamilton syringe and injected with BE (10 mg/kg) or FLC (10 mg/kg) at once. For virulence assays (10 larvae per group), all larvae were incubated at 37°C for 10 days. The death of G. mellonella was assessed daily and analyzed statistically by the Kaplan-Meier method (log-rank test).

### Crystallization of Eno1, data collection, structure determination, and refinement.

Crystals were grown at 13°C using the sitting drop vapor-diffusion method, with an initial mixing condition of 1 μL Eno1 (9.6 mg/mL) or Eno1-2PG solution (10 mg/mL) with an equal volume of reservoir solution. The initial crystallization conditions for Eno1 and the Eno1-2PG complex were determined using the crystal screen, index, and Proplex kit (Hampton Research). The best crystal of Eno1 was grown at 13°C from 0.2 M lithium sulfate monohydrate, 0.1 M Bis-Tris (pH 6.5), and 25% (wt/vol) polyethylene glycol 3350. In comparison, the Eno1-2PG crystal was obtained at 13°C in 0.2 M magnesium acetate tetrahydrate, 0.1 M sodium cacodylate trihydrate (pH 6.5), and 20% (wt/vol) polyethylene glycol 8000.

All crystals were soaked in cryoprotectant buffer containing 20% glycerol (vol/vol) and then flash cooled in liquid nitrogen. X-ray diffraction data were collected at the synchrotron radiation beamline BL17U1, Shanghai Synchrotron Radiation Facility (SSRF), using an Area Detector Systems Corporation (ADSC) Quantum 315R charge-coupled-device (CCD) detector with a crystal-to-detector distance of 300 nm for Eno1 and 200 mm for Eno1-2-PG. Diffraction data sets of Eno1 and Eno1-2PG were indexed, integrated, scaled, and merged using the program XDS (57) and HKL2000 (58), respectively. The crystals of Eno1 diffracted to 2.3 Å in space group C2 with unit cell parameters a = 265.20 Å, b = 62.18 Å, and c = 111.82 Å, and the crystal of Eno1 cocrystallized with 2-PG diffracted to 1.7 Å in space group C2 with unit cell parameters a = 264.264 Å, b = 61.848 Å, and c = 111.752 Å. Molecular replacement was performed by Phaser (47) in Phenix (48) using the 2AL2 structure of S. cerevisiae enolase for both Eno1 data sets (59, 60). Structure refinement, manual model building, and structural analysis were carried out using REFMAC5 (61) and WinCoot (62). The X-ray diffraction data processing and structure refinement statistics for both structures are summarized in the supplemental material (Table 1). The quality of the final models was validated using programs MolProbity and showed good stereochemistry. Figures were generated using PyMOL (http://www.pymol.org).

### Molecular docking.

Docking studies were based on the crystal structure of CaEno1 determined by our group and completed by Schrödinger Software Suite (63). Water molecules were first removed. After the addition, by standard protein preparation protocol, of missing hydrogen atoms, charges, and residues, the energy was minimized using the OPLS 2005 force field with root mean square deviation (RMSD) converged to 0.3 Å. The structure of BE was optimized, the docking of BE was carried out using Glide with a standard precision protocol, and the binding conformation of the lowest score was selected.
