# Supplementary material for: Baicalein Acts against Candida albicans by Targeting Eno1 and Inhibiting Glycolysis
Source: Microbiol Spectr. 2022 Jul 28;10(4):e02085-22. doi: 10.1128/spectrum.02085-22 (PMC9430770; doi:10.1128/spectrum.02085-22)
Supplement: Supplemental file 1 — Supplemental material. Download spectrum.02085-22-s0001.pdf, PDF file, 1.3 MB [file spectrum.02085-22-s0001.pdf]

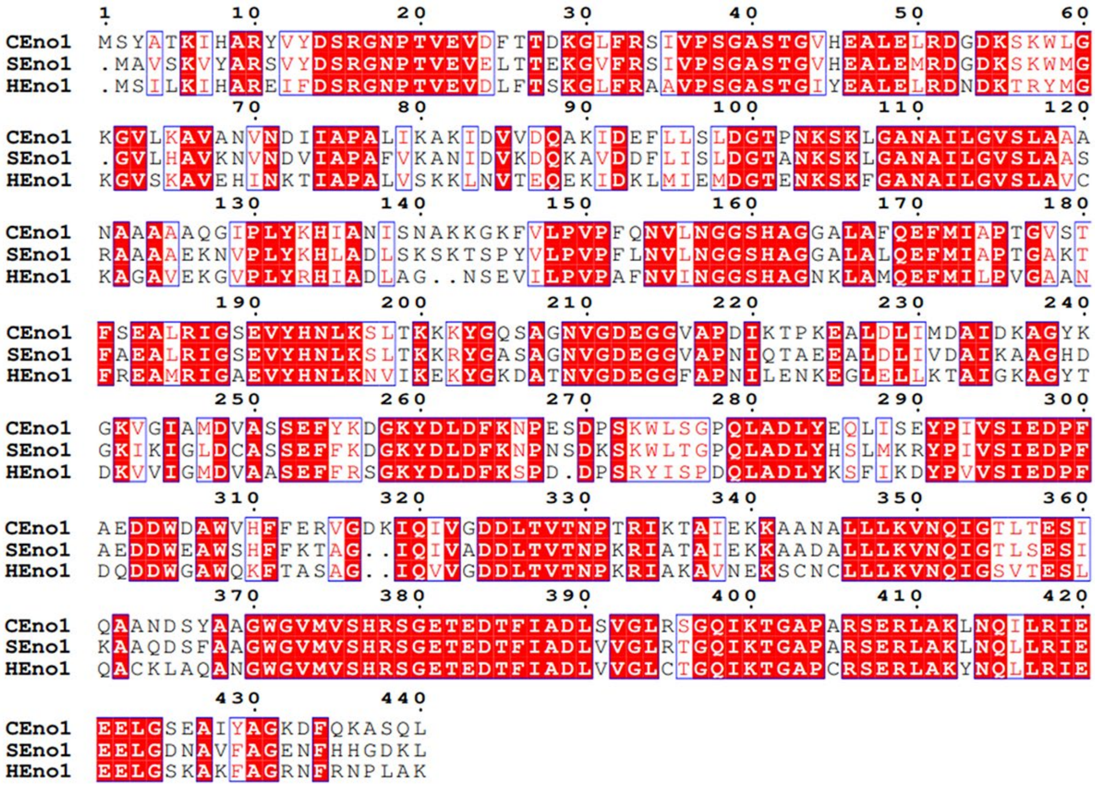

Figure S1. Alignment of amino sequences of *C. albicans* Eno1 with *S. cerevisiae* Eno1 and human Eno1. The identity of amino acid sequences of *C. albicans* Eno1 vs. *S. cerevisiae* Eno1 and *C. albicans* Eno1 vs. human Eno1 were 76.66% and 61.98%, respectively.

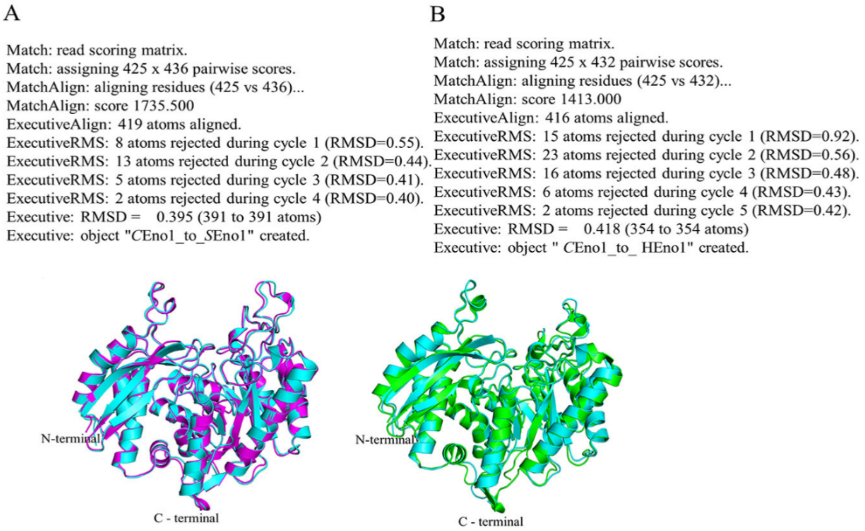

Figure S2. Comparative analysis of the structure of *C. albicans* Eno1 (blue) with *S. cerevisiae* Eno1 (purple) **(A)** and human Eno1 (green) **(B)**.

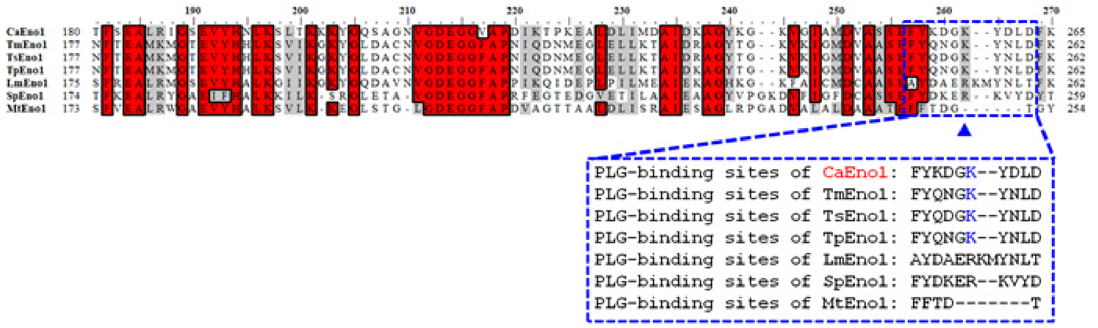

Figure S3. The multiple alignment analysis between *C. albicans* Eno1 and Eno1 with known PLG-binding sites (marked by a blue dotted line) from different kinds of micro-organisms. Ca *C. albicans*; Tm *Taenia multiceps*; Ts *Taenia solium*; Tp *Taenia pisiformis*; Lm *Leishmania mexicana*; Sp *Streptococcus pneumoniae*; Mt *Mycobacterium tuberculosis*.

Table S1A. Potential targets of BE obtained by a competition experiment of BE and TAMRA-N3 labeled label-BE

No.	Protein Name	Score (>700)	Coverage	Unique Peptides (>10)	Peptides	PSMs	Area	AAs	MW [kDa]	calc. pI
1	Acc1	11917.41	62.57	134	134	356	2.004E9	2271	253.3	6.40
2	Pyc2	9655.40	70.43	83	83	300	2.469E9	1177	129.7	6.38
3	Pdc11	2772.71	58.55	28	28	89	9.656E8	567	62.4	5.58
4	Adh1	2448.85	71.63	23	26	77	1.303E9	349	36.8	6.05
5	Tdh3	2225.91	80.00	26	26	61	1.289E9	335	35.8	7.12
6	Tef1	2047.35	52.62	27	27	72	1.610E9	458	50.0	9.03
7	Cdc19	1635.60	53.77	25	25	44	2.747E8	504	55.4	6.99
8	Pgk1	1457.30	64.99	29	29	45	2.834E8	417	45.2	6.48
9	Hsp70	1358.66	49.09	11	26	35	1.370E8	656	70.3	5.17
10	Eno1	1340.09	55.68	24	24	31	1.630E8	440	47.2	5.81
11	Cef3	1338.31	38.76	31	32	42	8.969E7	1050	116.9	5.69
12	Eft2	1272.14	43.94	34	34	43	2.736E8	842	93.3	6.47
13	Ssb1	1136.15	40.46	20	20	25	1.511E8	613	66.4	5.38
14	Gnd1	1044.79	50.30	22	22	28	1.174E8	495	54.3	6.09
15	Gad1	1014.33	42.25	17	17	23	4.677E7	568	63.9	6.55
16	Fba1	932.76	54.60	18	18	25	2.427E8	359	39.2	6.06
17	Pma1	923.09	27.15	19	19	25	9.121E7	895	97.6	4.98
18	Ald5	914.26	42.89	15	15	25	9.248E7	499	53.9	5.80
19	Rpl4B	902.12	46.83	13	13	21	2.579E8	363	39.2	10.74
20	Atp1	872.31	37.18	19	19	23	7.710E7	546	58.9	9.04
21	Tal1	857.20	55.11	16	16	20	4.658E7	323	35.7	5.00
22	Pfk1	791.39	27.36	21	23	28	3.724E7	987	108.5	6.62
23	Mdh1-1	784.22	59.34	12	12	16	4.501E7	332	34.7	6.10
24	Xyl2	747.14	46.11	13	13	18	3.800E7	360	38.7	5.97
25	Gph1	746.44	29.56	21	21	21	5.112E7	900	102.8	6.02
26	Fas1	746.39	13.45	22	22	23	1.893E7	2037	227.7	6.05
27	Atp2	746.10	43.65	13	13	17	5.699E7	504	53.9	5.19
28	Hsp90	716.07	30.27	18	18	20	2.850E7	707	80.8	4.88
29	Pck1	714.67	39.60	17	17	19	6.744E7	553	60.9	6.48

30	Pet9	700.16	43.52	16	16	19	2.744E8	301	32.7	9.60

Table S1B. Potential targets of BE obtained by a competition experiment of BE and biotin-N3 labeled label-BE

No.	Protein Name	Score (>700)	Coverage	Unique Peptides (>10)	Peptides	PSMs	Area	AAs	MW [kDa]	calc. pI
1	Adh1	9185.48	93.70	40	46	333	1.759E9	349	36.8	6.05
2	Pgk1	3507.30	93.53	52	53	111	2.451E8	417	45.2	6.48
3	Pyc2	1648.51	46.13	44	44	53	1.685E7	1177	129.7	6.38
4	Pda1	1422.99	66.58	27	27	44	1.466E7	401	44.1	8.13
5	Tuf1	1228.34	80.52	25	25	43	3.428E7	426	46.7	5.97
6	Erg10	1209.81	68.16	26	26	32	1.873E7	402	41.9	6.90
7	Acc1	1182.52	21.71	38	38	41	3.081E6	2271	253.3	6.40
8	Pdc11	1128.91	55.56	24	24	34	4.586E7	567	62.4	5.58
9	Gcv1	1054.49	64.72	20	20	32	4.142E7	394	43.7	7.61
10	Act1	969.20	67.82	22	22	32	2.520E7	376	41.7	5.69
11	Sam2	964.53	62.60	23	23	33	5.586E7	385	42.2	5.97
12	Gnd1	951.14	54.14	21	21	30	8.268E6	495	54.3	6.09
13	Eno1	918.49	46.82	20	20	23	5.501E6	440	47.2	5.81
14	Idp1	857.95	51.73	21	21	27	5.010E6	433	48.5	6.96
15	Tef1	771.26	58.30	23	23	28	2.103E7	458	50.0	9.03
16	Gln1	711.93	57.91	19	19	25	2.682E7	373	41.7	6.07

Table S1C. LC-MS/MS analysis showed that Eno1 was the dominant protein inbrightness changed band

No.	Protein Name	Score	Coverage	Unique Peptides	Peptides	PSMs	Area	AAs	MW [kDa]	calc. pI
1	Eno1	27983.96	80.00	41	41	695	6.362E10	440	47.2	5.81
2	Fdh3	57.30	3.14	1	1	1	1.549E7	255	27.4	5.69
3	Zcf22	40.44	1.34	1	1	1	9.970E8	448	50.6	5.96
4	Utp20	39.55	1.25	1	1	2	1.388E8	1197	136.6	5.66
5	Tuf1	38.46	3.52	1	1	1	1.033E8	426	46.7	5.97
6	Jem1	37.08	1.42	1	1	1	4.165E7	636	74.1	8.75

7	[illegible]O19.110	30.17	2.19	1	1	1	9.736E7	547	62.9	5.06
8	Rad54	29.95	1.06	1	1	1	1.963E9	848	95.6	8.95
9	Oct1	29.88	0.89	1	1	1	1.427E7	783	89.3	6.44
10	Atp1	29.77	1.90	1	1	1	5.950E6	368	39.7	9.42

Table S2A. Strains used in this study

No.	Strain	genotype	Source or reference
C. albicans			
1	SC5314	Wild type	Eukaryot Cell. 2005 Feb;4(2):298-309.
2	SN152	*his1 /his1 arg4 /arg4 leu2 /leu2*	Eukaryot Cell. 2005 Feb;4(2):298-309
3	*ENO1 /eno1*	*ENO1 /eno1 ::HIS1 arg4 /arg4 leu2 /leu2*	This study
4	*eno1 /eno1*	*eno1 ::HIS1 /eno1 ::ARG4 leu2 /leu2*	This study
E. Coli			
5	BL21(DE3)	F- ompT hsdSB(rB- mB-) gal dcm（DE3）	TIANGEN, China

Table S2B. Primers used in this study

No.	Primer na	Primer sequence (5'to3')
**Primers for construction of recombinant plasmid containing the *ENO1* gene**		
1	ENO1up	GGCCATATGATGTCTTACGCCACTAAAATC
2	ENO1dowr	CCGCTCGAGCAATTGAGAAGCCTTTTGG
**Primers for the *ENO1* gene deletion**		
3	ENO1 P1	CGGGAAGTTGTGTTCCATTC
4	ENO1 P3	cacggcgcgcctagcagcggTGTTGTAATATTCCTGAATT
5	ENO1 P4	gtcagcggccgcatccctgcGTTTGCCTCTGATTAAATAA
6	ENO1 P6	TCAGATTAGCCCCATTGCCG
7	Universal p	ccgctgctaggcgcgccgtgACCAGTGTGATGGATATCTGC
8	Universal p	gcagggatgcggccgctgacAGCTCGGATCCACTAGTAACG
**Primers for diagnosising the eno1/eno1 mutant strain**		
9	ENO1 Uch	GGTGATCCACACTAATTATA
10	ENO1 Dch	TCCAGCTGAAAGAGGATGTC
11	HIS1 Left	ATTAGATACGTTGGTGGTTC
12	HIS1 Right	AACACAACTGCACAATCTGG
13	ARG4 Left	ACACAGAGATACCTTGTACT
14	ARG4 Rigl	ACGGAGTACCACATACGATG

Table S3. Interaction of fluconazole (FLC) and BE probes against isolates of *Candida albicans 103* by MIC (μg/mL) of checkerboard microdilution assay.

Compounds	Molecular structure	Individual action MIC (μg/mL)	Interaction MIC(μg/mL)		FICI	Mode of interaction
			FLC	Compounds		
**BE**	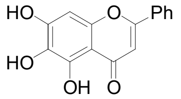	32	1	1	0.039	Synergism
**BE-1**	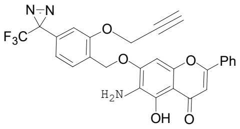	>64	>64	1	1.01	Indifference
**BE-2**	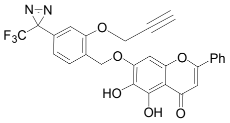	>64	1	4	0.039	Synergism
**BE-3**	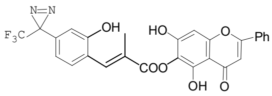	>64	1	4	0.039	Synergism
**BE-4**	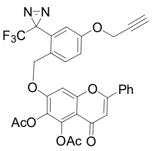	>64	>64	1	1.01	Indifference
**BE-5**	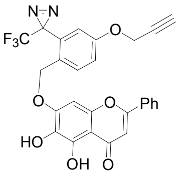	>64	0.25	8	0.0645	Synergism
**BE-6**	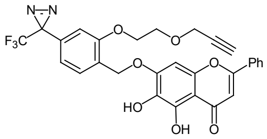	>64	>64	1	1.01	Indifference

Table S4. Structure and characterization of compounds were identified by NMR

No.	Name	Structural formula	Character	ESI-MS	NMR
1	TPD-6a	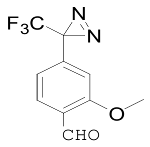	Yellow liquid	244.05	^{1}H-NMR(300MHz,$CDCl_3$):δ 10.45(s,1H),7.8(d,*J*=8.1Hz,1H),6.85(d,*J*=8.1Hz,1H),6.70(s,1H,),3.95(s,3H).
2	TPD-7a	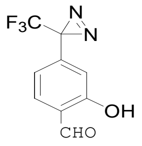	White solid	230.03	^{1}H-NMR(300MHz,DMSO-d_6): δ 11.15(s,1H),10.28(s,1H),7.72(d,*J*=6.4Hz,1H),6.87(s,1H),6.71(d,*J*=6.4Hz,1H)
3	TPD-8a	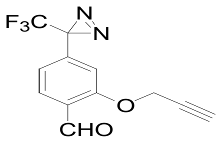	Yellow liquid	268.05	^{1}H-NMR(300MHz,$CDCl_3$): δ 10.44(s,1H),7.87(d,*J*=8.1Hz,1H),6.92(s,1H),6.87(d,*J*=8.1Hz,1H),4.85(d,*J*=2.1Hz,2H),2.63(t,*J*=2.1Hz,1H).
4	TPD-9a	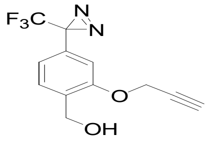	Light yellow liquid	270.06	^{1}H-NMR(300MHz,$CDCl_3$): δ 7.39(d,*J*=7.8Hz,1H),6.83(d,*J*=9.0Hz,2H),4.77(d,*J*=2.4Hz,2H),4.71(s,2H),2.58(t,*J*=2.4Hz,1H)
5	TPD-10a	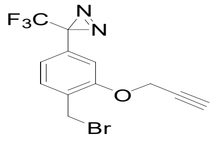	White solid	331.98	^{1}H-NMR(300MHz,$CDCl_3$): δ 7.39(d,*J*=8.1Hz,1H),6.83(s,1H),6.79(d,*J*=8.1Hz,1H),4.80(d,*J*=2.4Hz,2H),4.52(s,2H),2.58(t,*J*=2.4Hz,1H)
6	BE'	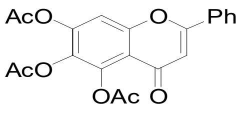	Grayish white solid	396.08	^{1}H-NMR(600MHz,$CDCl_3$):δ 7.87-7.85(m,2H),7.55-7.50(m,4H),6.66(s,1H),2.45(s,3H),2.35(s,3H),2.34(s,3H).
7	BE-1	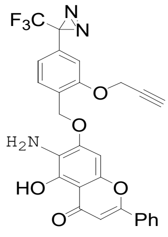	Yellow solid	521.12	^{1}H-NMR(600MHz,DMSO-d_6):δ8.56 (s,1H), 8.04 (d, *J* = 7.2 Hz, 2H), 7.73 (d,*J*=7.8Hz,1H),7.62-7.50(m,3H), 7.04 (d,*J*=7.8Hz,1H),6.97(s,1H),6.82(s,2H), 6.74(s, 1H),6.64(s,1H),5.24(s, 2H), 5.02 (s,2H),
8	label-BE	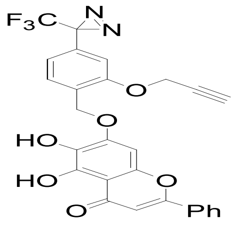	Yellow solid	522.1	^{1}H-NMR(600MHz,DMSO-d_6):δ 12.58(s,1H),8.83(s,1H),8.11(d,*J*=8.4Hz,2H),7.69(d,*J*= 8.4Hz,1H),7.60(m,3H),7.04(d,*J*=5.4Hz,2H),7.01(s,1H),6.98(s,1H),5.26(s,2H),5.01(d,J=2.4Hz,2

9	B	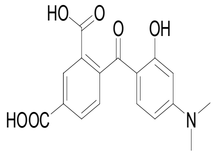	Purple powder	329.3	(DMSO-d_6, 300 MHz) δ : 13.44 (s, 2H), 12.38(s, 1H), 8.49 (d, 1H, J = 1.7 Hz), 8.20 (dd, 1H, J =7.9, 1.7 Hz), 7.52 (d, 1H, J = 7.9 Hz), 6.80 (d, 1H, J = 9.2 Hz), 6.20 (dd, 1H, [illegible]
10	C	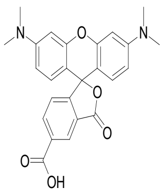	Purple powder	430.4	(CD_3OD-d, 300 MHz) δ: 8.75 (s, 1H), 8.18 (d, 1H, J = 7.5 Hz), 7.28 (d, 1H, J = 7.5 Hz), 7.25 (d, 2H, J = 9.3 Hz), 6.98 (dd, 2H, J = 9.3, 1.7 Hz), 6.83 [illegible]
11	TAMRA-N_3	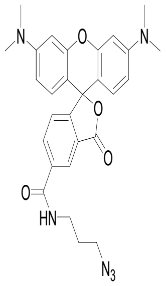	Purple powder	512.5	(DMSO-d_6, 300 MHz) δ: 8.85 (t, 1H, J = 5.4 Hz), 8.45 (s, 1H), 8.20 (d, 1H, J = 8.1 Hz), 7.30 (d, 1H, J = 7.8 Hz), 6.51 (s, 6H), 3.46-3.36 (m, 4H), 1.85-1.76 (m, 2H).
12	D	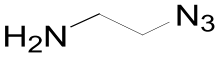	Colorless liquid	86.1	（$CDCl_3$, 300 MHz） δ: 3.30 (t, 2H, J = 5.7 Hz), 2.84-2.80 (m, 2H,), 1.27 (s, 2H)
13	E	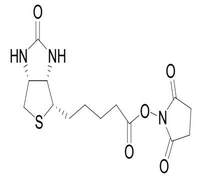	White solid	341.3	(DMSO-d_6 , 300 MHz) δ: 6.42 (s, 1H), 6.36 (s, 1H), 4.32-4.27 (m, 1H), 4.16-4.11 (m, 1H), 3.12-3.06 (m, 1H), 2.85-2.78 (m, 5H), 2.66 (t, 2H, J = 7.3 Hz), 2.57 (d, 1H, J = 11.4 Hz), 1.68-1.58 (m, 3H), 1.54-1.35 [illegible]
14	biotin-N_3	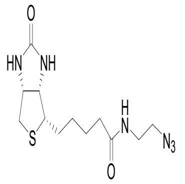	White solid	312.3	(DMSO-d_6, 300 MHz) δ: [illegible] (t, 1H, J = 5.4 Hz), 6.42 (s, 1H), 6.35 (s, 1H), 4.32-4.26 (m, 1H), 4.13-4.09 (m, 1H), 3.31 (d, 2H, J = 7.6 Hz), 3.24-3.19 (m, 2H), 3.11-3.05 (m, 1H), 2.80 (dd, 1H, J = 12.4, 5.1 Hz), [illegible]

Text S1

Synthesis of label-BE

Weigh m-bromoanisole (20 g, 106 mM) into a 500 ml three-neck flask, add 300ml of dry tetrahydrofuran (THF) for dissolution, protect with nitrogen, and slowly add n-hexane solution of n-butyl lithium (64 ml, 2.5 M in hexane, 1.5 eq) at -78 °C. After addition, stir for a reaction for 10 min, and then slowly drop F_3COOMe (18.2 g, 140 mM, 1.3 eq) at - 78 °C. After reaction for 2h, the reaction is complete by thin-layer chromatography (TLC). Dissolve 23.6 ml concentrated hydrochloric acid in 56.2 ml methanol, then add the solution to the reaction solution to quench the reaction. Raise the temperature to 0 °C, add 140 ml ethyl acetate (EA) to stir, and then use water (2 × 30 ml), dry with anhydrous sodium sulfate, spin dry the solvent to obtain the crude product of colorless liquid compound TPD-1 (17.5 g, yield 81 %), which is directly used in the next reaction.

Weigh TPD-1 (17.5 g, 86 mM) into a 250 ml round bottom flask, add 62 ml of absolute ethanol to dissolve, then add hydroxylamine hydrochloride (7.7 g, 112 mM, 1.3 eq), 125 ml of pyridine, reflux at 85 °C for 10h, detect the complete reaction by TLC. Spin the solvent dry under reduced pressure, add 100 ml EA, and then use water (3 × 100 ml) wash, 1M HCl (2 × 100 ml) wash with water (1 × 100 ml), dried with anhydrous sodium sulfate, and spin dry the solvent to obtain a colorless oily compound TPD-2 (17.5 g, yield 93%).

Dissolve TPD-2 (17.5 g, 80 mM) in pyridine (200 ml), add p-toluenesulfonyl chloride (22.9 g, 120 mM, 1.5 eq) in batches, reflux at 125 °C for 2 h, cool to room temperature, spin dry the solvent under reduced pressure, add 200 ml water, and then EA (3 × 100 ml) extraction, 1 M HCl (2 × 100 ml) washing water (2 × 100 ml) was washed, dried with anhydrous sodium sulfate, spin dry the solvent, recrystallized (Petroleum ether (PE): EA = 3:1) and purified to obtain solid white TPD-3 (21.9 g, yield 73%).

Dissolve TPD-3 (21.9 g, 58.7 mM) in CH_2Cl_2 (200ml), pass about 50 ml of liquid ammonia at - 78 ℃, raise the temperature to - 50 °C for a reaction for 10h, open it at room temperature overnight, make the excess ammonia absorb into water or discharge into the atmospheric environment, filter out impurities, and use water for filtrate (2 × 100 ml), dry with anhydrous sodium sulfate, spin dry the solvent to obtain the crude product of light yellow liquid compound TPD-4 (8.6 g, yield 67%).

Weigh TPD-4 (9.0 g, 41.3 mM) in a 100 ml round bottom flask, add methanol (50 ml) to dissolve it, then add triethylamine (8.4 g, 82.6 mM, 2 eq), slowly add I_2 (10.5 g, 41.3 mM) under ice bath conditions, react at room temperature for 3 h, TLC test the reaction is complete, add 20 ml of 10% citric acid solution, and then add sodium thiosulfate until the color of the reaction solution becomes lighter and EA (3 × 50 ml) extraction, water (3 × 50 ml), dry with anhydrous sodium sulfate, spin dry the solvent to obtain yellow liquid TPD-5 (8.0 g, yield 90%).

Place TPD-5 (8.0 g, 37 mM) in a 500 ml round bottom flask, add 200 ml CH_2Cl_2 to dissolve it, slowly add $TiCl_4$ (10 g, 55.5 mM, 1.5 eq) at 0 °C, then add Cl_2CHOMe (6.0 g, 55.5 mM, 1.5 eq), after adding, rise to room temperature for a reaction for 1 h, TLC test the reaction completely, add 250 ml water at 0 °C to quench the reaction, and then successively use water (2 × 100 ml) wash, saturated $NaHCO_3$ (2 × 100 ml) washing water (2 × 100 ml) washing, anhydrous sodium sulfate drying, spin dry the solvent, silica gel column chromatography (PE: EA = 100:1) to obtain yellow oily compounds TPD-6a (2.7 g, yield 30%).

Weigh TPD-6a (1.45 g, 6 mM) in a 50 ml round bottom flask, add 20 ml CH_2Cl_2 to dissolve, protect with nitrogen, and slowly drop BBr_3 solution (1.65 ml, 4 M in CH_2Cl_2) at - 20 °C. The drop was raised to 0 °C for 1 h, the reaction was complete by TLC detection, 20 ml water was added, and CH_2Cl_2 (3 × 20 ml), combine the organic layers, and use saturated sodium chloride (3 × 20 ml), dry with anhydrous sodium sulfate, spin dry the solvent to obtain solid white TPD-7a (1.1 g, yield 80%).

Weigh and dissolveTPD-7a (0.5 g, 2.17 mM) in 10 ml N,N-Dimethylformamide, successively add K_2CO_3 (0.6 g, 4.34 mM, 2 eq), bromopropargyne (0.258 g, 2.17 mmol, 1 eq), react at room temperature for 2 h, complete the reaction by TLC, add 10 ml water, and then EA (3 × 10 ml), and the organic layer is successively extracted with saturated sodium bicarbonate (2 × 10 ml) wash, saturated sodium chloride (2 × 10ml), dried with anhydrous sodium sulfate, evaporated and dried the solvent to obtain yellow liquid TPD-8a (0.57 mg, yield 98%).

Weigh TPD-8a (0.26 g, 1 mmol) into a 25 ml round bottom flask, add 6 ml THF and 0.2 ml water, then add $NaBH_4$ (20 mg, 0.5 mmol, 0.5 eq) into the reaction solution, react at room temperature for 2 h, complete the reaction by TLC, add 6 ml water, and then EA (3× 6 ml) and the organic layer was extracted with saturated sodium bicarbonate (2 × 6 ml) washing, saturated sodium chloride (2 × 6 ml), dried with anhydrous sodium sulfate, evaporated and dried the solvent to obtain light yellow liquid TPD-9a (0.23 mg, yield 85%).

Weigh TPD-9a (0.17 g, 0.63 mmol) in a 25 ml round bottom flask, add 2 ml CH_2Cl_2 for dissolution, protect with nitrogen, and drip PBr_3 (0.062 g, 0.23 mmol, 0.36 eq) at 0 °C, react at room temperature for 1 h, complete the reaction by TLC, add 2 ml water, CH_2Cl_2 (3 × 2ml) extraction, saturated sodium bicarbonate (3 × 2 ml) wash, saturated sodium chloride (3 × 2 ml), dried with anhydrous sodium sulfate, evaporated and dried the solvent to obtain solid white TPD-10a (0.18 g, yield 88%).

Weigh BE (1.35 g, 5 mmol) in 25 ml round bottom flask, add acetic anhydride (10 ml), sodium acetate (41 mg, 0.5 mmol, 0.1 eq), 80 °C for 2 h, thin-layer chromatography (TLC) detection reaction is complete, pour the reaction liquid to 50 ml ice water, filter, wash the cake with a small amount of cold ethanol, and drain the filter residue to obtain the gray-white solid BE’ (1.63 g, yield 82%). Take 25 ml of dried acetone into 50 ml round bottom flask, add BE’ (277 mg, 0.7 mmol), TPD-10a (700 mg, 2.1 mmol, 3 eq), K_2CO_3 (386 mg, 2.8 mmol, 4 eq), KI (11.62 mg, 0.07 mmol, 0.1 eq), 60 ℃, and react for 8 hours under nitrogen protection condition. After TLC detection, filter the hot

mixed solution and dry the filtrate. The crude product was washed with a small amount of cold EA and vacuum dried to obtain solid white BE-0 (310 mg, yield 73%). BE-0 (100 mg, 0.165 mmol) was dissolved with 2 ml methanol, 2 ml ammonia was added, heated, and reflux for 18 h, TLC detection reaction was complete, and the reaction was completely reduced to room temperature, 2 ml of water were added and EA (3 × 5ml) extraction, and washing by water (2 × 5 ml), drying the solvent, the crude product was obtained the yellow solid BE-1 (70 mg, yield 81%) was obtained by silica gel column chromatography (DCM: MeOH = 20:1). BE-0 (100 mg, 0.165 mmol) was dissolved with 5 ml ethanol, 0.3ml of concentrated hydrochloric acid was added, nitrogen was protected, and the reaction of TLC was complete for 10 hours. The yellow solid was obtained by vacuum concentration, filtered, washed with cold ethanol (5 ml), and the yellow solid label-BE (76 mg, yield 88%) was obtained by recrystallization of dichloromethane isopropyl ether system. The compounds were identified by NMR (Bruker DRX-600) (Table S6).

Synthesis of TAMRA-N_3

Synthesis of TAMRA-N_3 was performed as described previously . Weigh and dissolve sodium azide (1.95 g, 30 mmol, 3 eq) in H_2O (8 ml), slowly add chloropropylamine hydrochloride (1.29 g, 10 mmol), add it, react at 80 °C for 10 h, and detect that the reaction is complete; Cool the reaction liquid to 0 °C, add solid KOH (3.28 g), add ether (8 ml), separate the organic layer, and use ether (3 × 10 ml) for the water layer extraction, combined organic layer, anhydrous MgSO4, drying, filtration, spin-drying, crude product A (980 mg) was obtained. Weigh 3 - (dimethylamino) phenol (1.37 g, 10 mmol) into a round bottom flask, add anhydrous toluene (30 ml) to dissolve it, raise the temperature to 60 °C, add trimellitic anhydride (2.30 g, 12 mmol, 1.2 eq), add it, reflux at 120 °C for 24 hours. Cool the reaction liquid to room temperature, filter, and use toluene (3× 5 ml) for the filter cake washing; Dissolve the filter cake in MeOH (30 ml), reflux for 10 min, add formic acid (10 ml) and then spin dry the solvent. The obtained solid is dissolved in MeOH (20 ml). After refluxing for 2 h, cool the solution and place

it at 4 °C overnight; After filtration, the filter cake was washed with MeOH (5 ml) to obtain compound B (2.5 g, 76%). Compound B (1.3 g, 3.9 5 mmol) and 3 -(dimethylamino) phenol (0.65 g, 4.74 mmol) were added to dry DMF (50 ml), polyphosphoric acid (2.6 g) was added, heated to 120 °C for 8 hours; Cool the reaction solution to room temperature, pour it into 200 ml of distilled water, stir it overnight; After filtration, the filter cake was washed with ice water to obtain grayish-brown solid compound C (1.55 g, 91.1%). Dissolve compound C (0.5 g, 1.16 mmol) in DMF (30 ml), add PyBoP (0.605 g, 1.16 mmol, 1 eq), DIEA (0.30 g, 2.32 mmol, 2 eq), stir for 5 min, dissolve compound A (0.118 g, 1.16 mmol, 1 eq) in DMF (5 ml), add it into the reaction solution, stir at room temperature for 2 h; EA (50 ml) in the reaction solution was diluted with 5% $NaHCO_3$ (1 × 10 ml) wash, saturated NaCl (1 × 10 ml), dried with anhydrous Na_2SO_4, filtered, and evaporated the filtrate through the column to obtain grayish-brown solid compound TAMRA-N3 (450 mg, 75.6%). TAMRA-N_3 was identified by NMR (Bruker DRX-600) (Table S6).

Synthesis of biotin-N_3

Biotin-N_3 was synthesized by the following method. Weigh and dissolve sodium azide (1.904 g, 29.3 mmol, 3 eq) in H_2O (8 ml), slowly add bromoethylamine hydrobromate (2 g, 9.76 mmol), add it, raise the temperature of the reaction solution to 75 °C, stir for 21 h, and detect that the reaction is complete; Cool the reaction liquid to 0 °C, add solid KOH (3.2 g), add ether (8 ml), separate the organic layer, and use ether (3× 10 ml) for the water layer extraction, combined organic layer, anhydrous $MgSO_4$, drying, filtration, spin-drying, directly used in the next step. Crude product D (783 mg) was obtained. Weigh biotin (1.5 g, 6.14 mmol) into a round bottom flask, add DMF (50 ml) to dissolve, add n-hydroxysuccinamide (0.85 g, 7.37 mmol, 1.2 eq), EDC · HCl (1.41 g, 7.37 mmol, 1.2 eq), add it, stir at room temperature for 21 h, and the reaction is complete; Evaporate the solvent, add CH_2Cl_2 (200 ml), and use saturated $NaHCO_3$ (2 × 10 ml) for the organic phase wash, dried with anhydrous $MgSO_4$, filtered and spin-dried to obtain the white solid compound E (1.15 g, 55%). Weigh compound D (241 mg, 2.8 mmol, 1.4 eq) into

a round bottom flask, add DMF (30 ml) to dissolve, add Et_3N (0.3 ml, 2 mmol, 1 EQ), dissolve compound E (700 mg, 2 mmol) in DMF (20 ml), stir at room temperature for 24 hours. The solvent was evaporated dry, and the white solid compound biotin-N_3 (477 mg, 75%) was obtained by silica gel column chromatography (Table S6).
